# From 5G smart hospitals to 6G-enabled learning hospitals: a governance-oriented framework for healthcare technology, policy, and responsible deployment

**DOI:** 10.3389/fdgth.2026.1923445

**Published:** 2026-09-21

**Authors:** Guiyang Zhou, Lisha Wu

**Affiliations:** 1 Department of Medical Education Management, Shenzhen Bao’an Authentic TCM Therapy Hospital, Shenzhen, Guangdong, China; 2 General Administration Office, Longgang Central Hospital of Shenzhen, Shenzhen, Guangdong, China

**Keywords:** 6G-enabled learning hospitals, digital health governance, edge-cloud computing, evidence maturity, explainable artificial intelligence, internet of medical things, responsible deployment, smart hospitals

## Abstract

**Objectives:**

Discussion of 6G in healthcare often moves directly from anticipated network capabilities to proposed clinical applications, although application-level evidence, current connectivity alternatives, and 6G-specific evidence differ substantially in maturity. This narrative review develops an evidence-calibrated, governance-oriented framework for prospective 6G-enabled learning hospitals and examines when future 6G may add meaningful value beyond existing infrastructure.

**Methods:**

A multidisciplinary narrative synthesis covered smart hospitals, learning health systems, 5G/6G communication, IoMT, edge-cloud computing, AI/XAI, robotics, digital twins, cybersecurity, interoperability, implementation, and digital-health governance. Structured searches of PubMed, Web of Science, IEEE Xplore, and Google Scholar covered 1 January 2010–31 January 2026, supplemented by relevant standards, regulatory and policy sources, and targeted recent studies. Evidence was interpreted using an author-developed Evidence Maturity Classification (EMC) spanning demonstrated implementation, clinically grounded empirical, technical/benchmark, and prospective evidence.

**Results:**

The synthesis produced a seven-layer architecture, nine use-case analyses, a capability and evidence-maturity matrix, a twelve-domain risk-governance-metric framework with decision gates, a five-phase implementation roadmap, and an executive checklist. Across use cases, evidence for the underlying healthcare application was more mature than evidence for incremental benefit specifically attributable to 6G. Existing wired networks, Wi-Fi, private cellular systems, 5G/5G-Advanced, and edge-cloud infrastructure already support many monitoring, telemedicine, imaging, home-care, logistics, maintenance, and selected teleoperation functions. Future 6G is therefore most relevant when meaningful residual capability gaps remain, particularly combinations of mobility continuity, predictable end-to-end service, dense device coordination, multimodal synchronization, integrated sensing or positioning, and communication-computing orchestration.

**Conclusions:**

6G adoption should follow demonstration of clinical or operational value, a residual capability gap, and governance readiness. Hospitals should use the least complex infrastructure that meets requirements, generate evidence through risk-proportionate pilots, and scale advanced connectivity only when incremental value is demonstrated against optimized current alternatives. The framework provides a structured basis for these decisions but requires prospective validation across diverse hospital settings.

## Highlights

**What are the main findings?**

- Evidence supporting the underlying healthcare applications is more mature than evidence demonstrating an incremental benefit specifically attributable to 6G.
- The framework links a seven-layer architecture and nine use cases to evidence maturity, residual capability gaps, governance decision gates, and a five-phase implementation roadmap.

**What are the implications of the main findings?**

- Hospitals should optimize existing infrastructure and demonstrate a clinically or operationally meaningful residual capability gap before considering future 6G.
- Advanced connectivity should scale only when application value, safety, governance readiness, cost, equity, and sustainability have been demonstrated.

## 1 Introduction

Hospitals are moving beyond digitization toward increasingly connected and data-dependent care environments. Electronic health records, hospital information systems, picture archiving and communication systems, telemedicine, connected medical devices, cloud computing, and digital workflow platforms have changed how information is generated and exchanged. More recent smart-hospital programmes extend this infrastructure through IoMT, artificial intelligence, robotics, automation, remote monitoring, and operational analytics (1–6). Yet greater digital density does not necessarily create a learning hospital. The defining challenge is whether information generated during care can be converted into reliable decisions, evaluated against meaningful outcomes, and fed back into subsequent clinical and organizational improvement (1–3).

Communication infrastructure is one component of this transition. Fifth-generation mobile networks have expanded the technical options available for mobile telemedicine, connected devices, high-bandwidth data transfer, edge computing, and differentiated services. Importantly, some applications frequently invoked in discussions of future 6G healthcare are already technically or clinically feasible using current networks. Field evaluation has demonstrated bidirectional audiovisual and mobile-ultrasound transmission between an ambulance and hospital over a 5G system (7). Selected robotic telesurgical procedures have also progressed from feasibility demonstrations to prospective clinical comparison using 5G connectivity (8). These studies remain limited in scale and setting, but they establish an important baseline: the existence of an advanced healthcare application does not, by itself, demonstrate a requirement for 6G.

6G should therefore be considered against the actual maturity of next-generation communication systems. The International Telecommunication Union's IMT-2030 framework identifies future usage scenarios and capabilities extending beyond IMT-2020, including highly reliable low-latency communication, massive communication, ubiquitous connectivity, AI and communication, and integrated sensing and communication (9, 10). General 6G research further discusses higher capacity, advanced spectrum use, distributed intelligence, sensing, positioning, and more adaptive coordination of communication and computing resources (11–15). These sources define technical trajectories and performance objectives; they do not constitute evidence that those capabilities are routinely available in hospitals or that they improve patient outcomes. Current standardization remains under development, with 3GPP Release 20 undertaking early 6G studies and normative specification work associated with Release 21 (16). Recent healthcare-oriented 6G publications remain predominantly reviews, perspectives, architecture studies, and engineering evaluations rather than mature clinical deployments (17–19). Claims regarding 6G healthcare should consequently remain prospective unless supported by system-level and clinical validation.

This distinction exposes a recurring problem in the 6G-healthcare literature. Technical capabilities are often mapped directly to compelling scenarios—remote surgery, critical-care monitoring, emergency coordination, high-resolution imaging, pervasive sensing, or autonomous systems (17–19)—without first establishing whether the underlying application is evidence-supported, whether current infrastructure already satisfies its requirements, or which clinically meaningful limitation would actually be removed by future 6G. These are separate questions. A use case can have mature clinical evidence while its proposed 6G component remains speculative; conversely, a technically credible network capability can exist without a validated clinical pathway.

The same separation is necessary for the other technologies expected to form future connected hospitals. IoMT already supports continuous and distributed sensing, but increasing device numbers does not resolve data quality, calibration, interoperability, or clinical interpretation (20–23). Edge-cloud architectures can reduce dependence on centralized processing and support latency-sensitive or privacy-sensitive workloads (24–26), yet the location of computation should be determined by the requirements of the clinical workflow rather than by architectural fashion. AI has demonstrated substantial potential in diagnosis, risk prediction, imaging, monitoring, and operational decision support (27, 28), while reporting and evaluation frameworks increasingly emphasize prospective clinical validation and transparent implementation (29–31).

Recent XAI research further illustrates both technical progress and the remaining translational gap. Explainable AI is increasingly being integrated with IoMT, multimodal data, and clinical prediction systems (32–35). Patient-level studies in sepsis show how explainability can assist interpretation of multivariable models, while also demonstrating that *post-hoc* explanation does not establish model validity and that greater model or biomarker complexity does not necessarily produce greater clinical utility (36, 37). These observations are directly relevant to future learning hospitals: AI, IoMT, robotics, or multimodal analytics can strengthen an application layer, but evidence supporting these technologies should not be transferred automatically to the connectivity layer. Demonstrating useful XAI is not evidence that 6G is required to deliver it.

Governance therefore becomes a central design problem rather than an adjunct to connectivity. Greater device density, distributed computing, automated decision support, and cyber-physical systems expand the consequences of failures in cybersecurity, privacy, interoperability, data quality, and network resilience (38–48). Robotics introduces additional requirements for physical safety, human override, training, and responsibility (49–52). Digital twins and hospital command systems depend on model fidelity and organizational capacity to act on information rather than data availability alone (53). More broadly, socio-technical and implementation research has shown that healthcare technologies may fail despite technical feasibility because of poor workflow fit, inadequate user preparation, uncertain value, organizational complexity, or difficulty sustaining implementation at scale (54, 55).

Cost, equity, and environmental sustainability further complicate the assumption that technologically advanced infrastructure is necessarily desirable. Future communication networks, edge nodes, cloud workloads, sensors, AI systems, and robotics entail capital, maintenance, workforce, energy, and lifecycle costs. Home-based and regional digital services can improve access, but they can also deepen inequalities when participation depends on connectivity, devices, digital literacy, or institutional resources (56). Responsible digital transformation therefore requires evidence not only that a technology can function, but that it can generate durable value without disproportionate clinical, financial, social, or environmental burden.

Taken together, the literature suggests that the central unresolved question is no longer whether future 6G systems could support healthcare applications. A more useful question is when additional 6G capability would be clinically or operationally justified after existing infrastructure has been optimized. Addressing this question requires three distinctions that are insufficiently explicit in much of the current discussion: application-level evidence vs. connectivity-specific evidence; current capability vs. residual capability gap; and technical feasibility vs. governance readiness. Without these distinctions, hospitals risk treating network generation as a proxy for digital maturity and technology adoption as a proxy for clinical progress.

This narrative review therefore conceptualizes the prospective 6G-enabled learning hospital as an evidence-calibrated socio-technical ecosystem rather than a connectivity-upgraded facility. The framework is built around a simple decision sequence: define a meaningful clinical or operational problem; assess the maturity of evidence supporting the application; establish what optimized existing infrastructure can already provide; identify any remaining capability gap; determine whether future 6G could plausibly address that gap; and apply risk-proportionate governance and evaluation before scaling.

The article makes four linked contributions. First, it develops a seven-layer learning-hospital architecture connecting clinical scenarios, IoMT sensing, heterogeneous connectivity, edge-cloud computing, AI/XAI decision support, robotics and automation, and embedded governance. Second, it introduces an Evidence Maturity Classification and applies it across nine representative hospital use cases to distinguish application maturity from evidence supporting an incremental 6G requirement. Third, it develops a risk-governance-metric framework in which cross-cutting risks are linked to safeguards, measurable indicators, and non-compensatory decision gates. Finally, it translates these principles into a five-phase implementation roadmap and an executive checklist (Box 1) that prioritize current-infrastructure optimization, evidence-generating pilots, residual capability-gap assessment, and selective rather than universal 6G scaling.

This framing changes the endpoint of the discussion. A successful future hospital should not be defined by how many applications use 6G. It should be defined by whether each layer of its digital infrastructure contributes demonstrable clinical or operational value, whether failures are governable, and whether the institution can learn from deployment and revise its systems accordingly.

Box 1 **Executive checklist for hospital leaders considering advanced connectivity or 6G-enabled applications.**

1. **Is there a clearly defined clinical or operational problem?** The application should address a specific, measurable problem and have a defined value pathway before technology selection.

2. **Is the underlying application sufficiently evidence-supported for its level of risk?** Applications supported mainly by conceptual, simulation, prototype, or benchmark evidence should not be treated as clinically mature; higher-risk uses require stronger evidence.

3. **Can the problem already be addressed using optimized existing infrastructure?** Assess **wired** networks, enterprise Wi-Fi, private cellular systems, 5G/5G-Advanced, current IoT, and edge-cloud computing before introducing a new network generation.

4. **Does a clinically or operationally meaningful residual capability gap remain?** Specify the unmet requirement, such as mobility continuity, predictable end-to-end service, device **density**, multimodal synchronization, sensing/positioning, or distributed computing.

5. **Are the relevant governance and lifecycle conditions in place?** Map the activated risks, **including** cybersecurity, privacy, interoperability, data quality, network reliability, AI accountability, robotic safety, human oversight, workforce, regulation, cost, equity, and sustainability.

6. **Have meaningful evaluation endpoints and decision criteria been defined?** Link **technical** performance to clinical or operational outcomes and specify criteria for modification, pause, withdrawal, and scale-up before deployment.

7. **Has the application demonstrated enough value to justify scaling?** Require reproducible **benefit**, acceptable risk, validated fallback, organizational readiness, and—where 6G is proposed—incremental value over optimized current alternatives.

**Decision rule.** Do not proceed to large-scale investment or 6G-specific deployment when a safety-critical question remains unanswered or when the answer to Questions 1–6 is “No” or “Unclear.”

**Learning-hospital principle.** Post-deployment evidence should support four possible decisions: continue, modify, pause, or withdraw.

## 2 Methods: narrative synthesis and framework development

This study was designed as a narrative review and conceptual framework article to examine how emerging communication, computing, artificial intelligence, automation, and governance capabilities may be integrated at the hospital level to support the transition from 5G-enabled smart hospitals toward prospective 6G-enabled learning hospitals. The objective was not to estimate the clinical effectiveness of 6G-enabled interventions or to provide an exhaustive systematic evidence synthesis. Rather, the review aimed to integrate relevant technical, clinical, organizational, implementation, and governance evidence into a hospital-level framework for future research, pilot deployment, policy development, and strategic planning.

Given the early and heterogeneous nature of 6G-specific healthcare evidence, which currently spans telecommunications engineering, digital health, clinical artificial intelligence, IoMT, robotics, cybersecurity, implementation science, hospital management, and health policy, a narrative synthesis was considered more appropriate than a formal systematic review or meta-analysis for the present objective. PRISMA-based study selection, formal risk-of-bias assessment, quantitative meta-analysis, and evidence-certainty grading were therefore not undertaken. Nevertheless, the revised manuscript describes the literature-identification strategy, evidence roles, and framework-construction process to improve methodological transparency.

### 2.1 Review design and analytical scope

The article was positioned as a governance-oriented narrative review combined with conceptual framework development. The hospital was treated as a socio-technical system rather than as a collection of isolated technologies. Accordingly, the synthesis considered not only anticipated 6G communication capabilities but also the clinical and operational scenarios in which such capabilities might generate value; the sensing, data, and computing infrastructure required to support these scenarios; the roles of AI, XAI, robotics, and automation; and the governance mechanisms necessary for safe, accountable, equitable, and sustainable implementation.

Three assumptions guided the analytical approach. First, 6G should not be interpreted simply as a faster wireless network. Instead, anticipated 6G capabilities were examined as potential components of a broader infrastructure for real-time sensing, differentiated connectivity, distributed edge-cloud intelligence, and clinical-grade coordination. Second, 6G was not assumed to be necessary for all smart-hospital applications. Existing wired networks, Wi-Fi, private cellular networks, 5G, and conventional edge-computing architectures may remain sufficient for many current hospital functions. Third, technical feasibility was considered necessary but insufficient for hospital implementation. Clinical validation, patient safety, cybersecurity, privacy, interoperability, AI accountability, human oversight, robotic safety, workflow integration, organizational readiness, cost-effectiveness, equity, and environmental sustainability were considered integral components of technology evaluation.

Throughout the synthesis, evidence was interpreted according to four maturity categories: EM1, demonstrated clinical or operational feasibility; EM2, clinically grounded empirical evidence; EM3, technical, benchmark, simulation, or prototype evidence; and EM4, prospective, standards-defined, or conceptual evidence. This Evidence Maturity Classification indicates proximity to real-world hospital implementation rather than formal methodological quality or certainty of evidence.

### 2.2 Literature identification and update strategy

The conceptual synthesis was supported by structured database searches in PubMed, Web of Science, IEEE Xplore, and Google Scholar. These searches were supplemented by relevant standards and regulatory websites, backward reference checking, and selective forward citation tracking. During revision, targeted supplementary identification was used to incorporate directly relevant recent application and implementation studies.

The structured database-search window extended from 1 January 2010 to 31 January 2026. This period was selected to capture the modern development of smart hospitals, learning health systems, socio-technical health-information infrastructure, IoMT, edge-cloud computing, clinical AI, and digital-health governance. Because dedicated 6G research emerged later, 6G-specific database searches focused primarily on literature published from 2019 to 31 January 2026. Earlier seminal publications were retained when necessary to establish foundational concepts or frameworks; targeted supplementary identification during revision allowed directly relevant newer studies to be considered separately from the structured database-search window.

Search concepts were organized around future connectivity; smart hospitals and learning health systems; IoMT and connected medical devices; edge-cloud and distributed computing; AI, XAI, multimodal analytics, and federated learning; robotics, teleoperation, automation, and digital twins; and cybersecurity, privacy, interoperability, governance, implementation, equity, workforce, cost, and sustainability. The supplementary revision-stage identification additionally focused on recent studies relevant to explainable AI and clinical or hospital implementation evidence for 5G telemedicine, telesurgery, predictive maintenance, logistics, infection control, command centers, and digital twins.

### 2.3 Eligibility, source prioritization, and evidence interpretation

Sources were considered relevant when they contributed directly to at least one component required for development of the hospital-level framework. Eligible source types included peer-reviewed empirical studies, systematic and scoping reviews, major narrative or technical reviews, foundational conceptual papers, international standards, regulatory guidance, professional guidance, and authoritative policy or governance documents.

Sources were excluded from substantive synthesis when they consisted primarily of commercial or promotional claims, news reports, duplicated publications, or generic technology discussions with no meaningful relevance to healthcare or to a technical capability required by the framework. General 6G engineering studies were retained selectively and used only to characterize anticipated communication capabilities, standards, enabling technologies, or performance objectives.

Evidence was interpreted according to the type of claim being supported. Standards and authoritative telecommunications frameworks were prioritized for future network objectives and technology trajectories; healthcare-specific empirical studies and reviews for clinical applications and workflow claims; regulatory guidance for safety, privacy, cybersecurity, and accountability; and socio-technical and implementation literature for organizational readiness, adoption, scale-up, and sustainability (1–6, 9–56). When direct 6G hospital evidence was unavailable, evidence from adjacent technologies—including 5G, private networks, IoMT, edge-cloud computing, clinical AI, XAI, federated learning, robotics, and digital twins—was used to assess technical plausibility and governance needs, without interpreting that evidence as proof that 6G itself is clinically necessary or superior.

### 2.4 Conceptual domains of the synthesis

The literature was organized into four interrelated domains: smart hospitals, learning health systems, and digital-health infrastructure (1–6); wireless communication and 6G-enabling technologies (9–19); IoMT, edge-cloud computing, AI/XAI, federated learning, robotics, automation, and digital twins (20–37, 49–53); and governance, cybersecurity, ethics, regulation, implementation, hospital management, workforce readiness, equity, and sustainability (38–56). These domains were synthesized according to their role within a hospital-level socio-technical system rather than treated as independent evidence streams.

### 2.5 Development and provenance of the design principles

Five integrative design principles guided framework development: scenario-driven design, layered but non-linear architecture, capability matching, embedded governance, and evaluation orientation. These principles were developed by the authors through integrative interpretation of learning-health-system concepts, socio-technical models, implementation science, digital-health governance, and healthcare-technology evaluation (1, 2, 54, 55). They were not derived through formal qualitative coding, thematic saturation, Delphi consensus, or quantitative evidence grading.

### 2.6 Framework construction and use-case mapping

Framework construction proceeded in four stages. First, the prospective 6G-enabled learning hospital was defined as a socio-technical ecosystem in which real-time sensing, differentiated connectivity, edge-cloud intelligence, explainable AI, selected automation, and embedded governance contribute to continuous cycles of sensing, analysis, decision support, action, evaluation, and institutional learning.

Second, a seven-layer architecture was developed to organize the principal functional components of this ecosystem. Third, the architecture was translated into a clinical use-case capability matrix spanning nine representative applications with different clinical value, latency sensitivity, bandwidth demand, reliability requirements, device density, automation intensity, and governance risk. Fourth, a risk-governance-metric framework and five-phase implementation roadmap were developed.

The use cases were mapped qualitatively according to residual capability gap and potential incremental value of future 6G, and clinical or operational risk and governance intensity. Positioning was based on the narrative synthesis and author judgment. No validated numerical scoring system, statistical weighting procedure, or formal consensus method was used; the mapping is therefore a heuristic decision-support visualization rather than a quantitative ranking.

### 2.7 Scope and limitations of the narrative synthesis

This review does not establish that 6G-enabled hospital applications are clinically effective. Direct evidence from mature, routine, real-world 6G hospital deployments remains limited, and many proposed use cases are conceptual, simulation-based, prototype-level, or extrapolated from adjacent technologies. General 6G engineering and standardization literature was necessary to characterize anticipated capabilities but should not be interpreted as healthcare-specific validation.

Because this was a narrative rather than systematic review, literature selection, prioritization, and conceptual interpretation involved author judgment. No formal risk-of-bias assessment, meta-analysis, or certainty-of-evidence grading was performed. The proposed architecture, EMC, use-case matrix, qualitative mapping, governance framework, and roadmap are conceptual outputs rather than validated clinical instruments and require prospective testing across diverse hospital settings.

## 3 Results: an evidence-calibrated framework for prospective 6G-enabled learning hospitals

### 3.1 From 5G smart hospitals to prospective 6G-enabled learning hospitals

Hospitals have progressively evolved from digitized information environments toward connected systems incorporating electronic health records, medical imaging, telemedicine, IoMT, artificial intelligence, automation, and data-driven operational management (1–6). This development has increased the availability and timeliness of information, but connectivity alone does not create a learning hospital. The more consequential question is whether clinical and operational data can be converted into reliable decisions, linked to measurable outcomes, and used to improve subsequent care and organizational performance (1–3).

The transition from a 5G-enabled smart hospital to a prospective 6G-enabled learning hospital should therefore be understood primarily as a conceptual and organizational transition rather than a network replacement. A smart hospital emphasizes connected services and increasingly real-time digital workflows; a learning hospital additionally requires continuous feedback between sensing, analysis, decision support, action, outcome evaluation, and institutional adaptation. Future 6G may support selected parts of this cycle, but it is neither sufficient nor universally necessary.

#### 3.1.1 From connectivity to institutional learning

Existing smart hospitals remain constrained by fragmentation across EHRs, imaging, laboratory platforms, bedside devices, pharmacy systems, operational technologies, and external services. Faster communication does not resolve inconsistent terminology, poor interoperability, incomplete data, or weak workflow integration (45, 48, 54). Similar limitations apply to AI and automation: technically capable systems may have little clinical influence when they are poorly integrated into decision pathways or lack adequate validation, human oversight, and organizational ownership (27, 29–31).

Governance becomes increasingly important as digital systems assume a larger role in care delivery. Greater device density and automated decision support expand risks related to cybersecurity, privacy, data quality, accountability, resilience, cost, and unequal access (38, 39, 46). A hospital can therefore be technologically advanced while remaining unable to determine whether its digital systems are safe, effective, sustainable, or clinically useful.

In this review, a prospective 6G-enabled learning hospital is defined as a socio-technical hospital ecosystem in which clinical and operational needs guide sensing, heterogeneous connectivity, distributed computation, AI-supported interpretation, selected automation, and governance within a continuous learning cycle. Its defining feature is not the presence of 6G itself, but the capacity to connect data, decisions, actions, outcomes, and learning and to modify technologies, workflows, and policies when evidence changes (1–3).

This distinction shifts evaluation from technology adoption to value. Relevant questions become whether monitoring reduces delayed recognition of deterioration, whether predictive maintenance reduces service interruption, whether AI changes useful clinical decisions, whether automation improves workflow without introducing new hazards, and whether digital services improve access without worsening inequity.

#### 3.1.2 What future 6G may add—and what it does not replace

Future 6G systems are expected to extend current communication capabilities through combinations of highly reliable communication, dense device coordination, advanced mobility support, integrated sensing and positioning, distributed intelligence, and more adaptive orchestration of communication and computing resources (9–19). These capabilities could become relevant in demanding hospital scenarios involving mobile multimodal data, high-density IoMT, distributed edge intelligence, advanced teleoperation, or continuously synchronized hospital systems.

Their potential value should nevertheless be interpreted against the current technological baseline. Wired infrastructure, enterprise Wi-Fi, private cellular networks, 5G/5G-Advanced, and edge computing can already support many remote consultation, monitoring, imaging, home-care, automation, and selected teleoperation functions (7, 8, 57–59). Consequently, the presence of an advanced healthcare application does not demonstrate a requirement for 6G.

The appropriate comparison is therefore optimized existing capability vs. the residual requirement of a defined workflow. Future 6G becomes relevant only when an application retains a clinically or operationally meaningful limitation after existing alternatives have been reasonably optimized. Such limitations may involve predictable end-to-end service, mobility continuity, synchronization of multiple high-volume streams, very dense mobile-device environments, integrated sensing or positioning, or large-scale coordination between communication and distributed computing.

This framing is also consistent with the maturity of the evidence. Current 6G standards, architectures, healthcare models, and implementation pathways remain under development, and much of the 6G-specific evidence is still engineering, prototype, standards-oriented, or conceptual rather than clinical (9–19, 57–64). The prospective 6G-enabled learning hospital should therefore be treated as a target-state model requiring progressive validation, not as a description of a currently established hospital type.

Table 1 summarizes this transition from digital hospitals to 5G-enabled smart hospitals and prospective 6G-enabled learning hospitals, including differences in data use, connectivity, computation, intelligence, automation, governance, evidence maturity, and evaluation logic.

**Table 1:** Conceptual transition from digital hospitals to prospective 6G-enabled learning hospitals.

Dimension	Digital hospital	5G-enabled smart hospital	Prospective 6G-enabled learning hospital target state	Implication for the proposed framework
Core logic	Digitization of clinical and administrative information	Connectivity and service extension through mobile networks, IoT, cloud platforms, and selected edge services	Continuous sensing, distributed intelligence, differentiated connectivity, responsible automation, and institutional learning	Progress from information digitization toward measurable learning and improvement rather than network replacement alone
Strategic aim	Improve information availability and reduce paper-based workflows	Enable connected care, mobile services, remote consultation, smart wards, and selected real-time applications	Create an adaptive clinical ecosystem capable of sensing, analysing, acting, evaluating, and improving continuously	Link 6G to defined clinical or operational capability gaps and measurable value
Data model	Data are recorded, stored, and retrieved	Data are increasingly connected, transmitted, and partially integrated	Data are prospectively envisioned to be continuously captured, processed, interpreted, and fed back into clinical and governance processes	Support a data-to-decision-to-action-to-learning cycle, conditional on data quality and interoperability
Connectivity requirement	Predominantly wired networks and local information systems	Wired networks, Wi-Fi, private cellular networks, 5G/5G-Advanced, and edge connectivity support many current applications	Future 6G may add value for selected scenarios requiring combinations of high reliability, mobility, dense connectivity, multimodal transmission, integrated sensing, and differentiated service assurance	Benchmark requirements against optimized existing infrastructure before claiming a 6G requirement
Computing architecture	Centralized servers and departmental systems	Cloud platforms with increasing deployment of edge computing	Hybrid and distributed edge-cloud intelligence for latency-sensitive, privacy-sensitive, and large-scale analytics	Determine processing location from latency, privacy, resilience, and computational requirements rather than network generation
Intelligence model	Limited analytics and retrospective reporting	Increasing use of AI for diagnosis, monitoring, scheduling, prediction, and resource management	Explainable, monitored, auditable, and governed AI integrated into continuously evaluated workflows	Make XAI, calibration, external validation, human oversight, and model monitoring core requirements
Automation model	Limited workflow digitization and rule-based automation	Selected logistics, pharmacy, robotic, and smart-device automation	Risk-stratified integration of AI, IoMT, robotics, distributed computing, and human supervision	Progress according to demonstrated value and clinical risk rather than technological sophistication
Governance focus	Information management, data protection, and basic compliance	Device management, cybersecurity, interoperability, workflow integration, and emerging AI governance	Cybersecurity, privacy, AI accountability, robotic safety, resilience, cost, equity, sustainability, and continuous evaluation embedded across the system	Design governance before deployment and link it to explicit decision gates
Evaluation logic	Adoption, information availability, and process digitization	Connectivity performance, service expansion, workflow efficiency, and user experience	Clinical outcomes, patient safety, resilience, trustworthiness, cost-effectiveness, equity, sustainability, and technical performance	Interpret technical indicators together with patient-, workflow-, organizational-, and governance-level outcomes
Evidence maturity	Predominantly EM1: mature and routinely deployed digital foundations	EM1–EM2 for selected applications: 5G and related technologies have demonstrated clinical or field feasibility in some settings	Predominantly EM3–EM4 for 6G-specific claims: engineering, prototype, standards-development, and conceptual evidence dominate	Report evidence maturity separately from technological ambition
Main limitation	Fragmented systems and limited intelligence	Connectivity does not automatically resolve interoperability, validation, governance, or workflow problems	High complexity, uncertain incremental benefit over optimized existing infrastructure, immature 6G-specific evidence, regulatory uncertainty, and implementation cost	Use a capability-matched, evidence-generating, staged implementation strategy
Typical maturity state	Established digital foundation	Current and evolving smart-hospital infrastructure	Prospective target state requiring progressive validation rather than a currently established hospital model	Progress through readiness assessment, capability-gap analysis, pilots, decision gates, and responsible scaling

The prospective 6G-enabled column describes a target-state conceptualization rather than currently validated or routinely deployed hospital capabilities. Several functions may already be supported by wired networks, Wi-Fi, private cellular networks, 5G/5G-Advanced, and current edge-cloud architectures. The conceptual transition draws on learning-health-system, smart-hospital, future-connectivity, and socio-technical implementation literature (1, 9, 19, 55).

#### 3.1.3 Implications for architecture and implementation

Three implications follow from this conceptual transition. First, architecture should be scenario-driven: clinical and operational problems should define requirements before a network, AI model, robotic platform, or computing architecture is selected. Second, deployment should be capability-matched: hospital applications differ in latency tolerance, bandwidth, mobility, device density, safety consequence, computational requirements, and governance intensity, and the least complex infrastructure capable of meeting those requirements should remain the initial benchmark. Third, implementation should be evidence- and governance-led: technical performance must be linked to clinical or operational outcomes, and scaling should depend on demonstrated value, acceptable risk, organizational readiness, and explicit decision gates rather than technology availability alone.

These principles provide the basis for the seven-layer architecture developed in the following section.

### 3.2 A seven-layer architecture for prospective 6G-enabled learning hospitals

The proposed architecture treats the learning hospital as an interconnected socio-technical system rather than a conventional technology stack. It consists of seven analytically distinct but operationally interdependent layers: clinical and operational applications; IoMT sensing and devices; heterogeneous connectivity; edge-cloud computing; AI/XAI and decision support; robotics and automation; and governance, risk, and sustainability.

The layers do not form a one-directional pipeline. Clinical needs determine what information is required; sensing systems generate observations; connectivity and computing determine where and when those observations become available; AI and other analytical methods support interpretation; automation translates selected decisions into action; and governance constrains every stage. Outcomes are fed back into system configuration, clinical workflows, model development, resource allocation, and institutional policy. Figures 1, 2 summarize the architecture and its iterative data-value and feedback flows; the text below therefore focuses on the function and principal design boundary of each layer rather than reproducing the figures.

**Figure 1:**
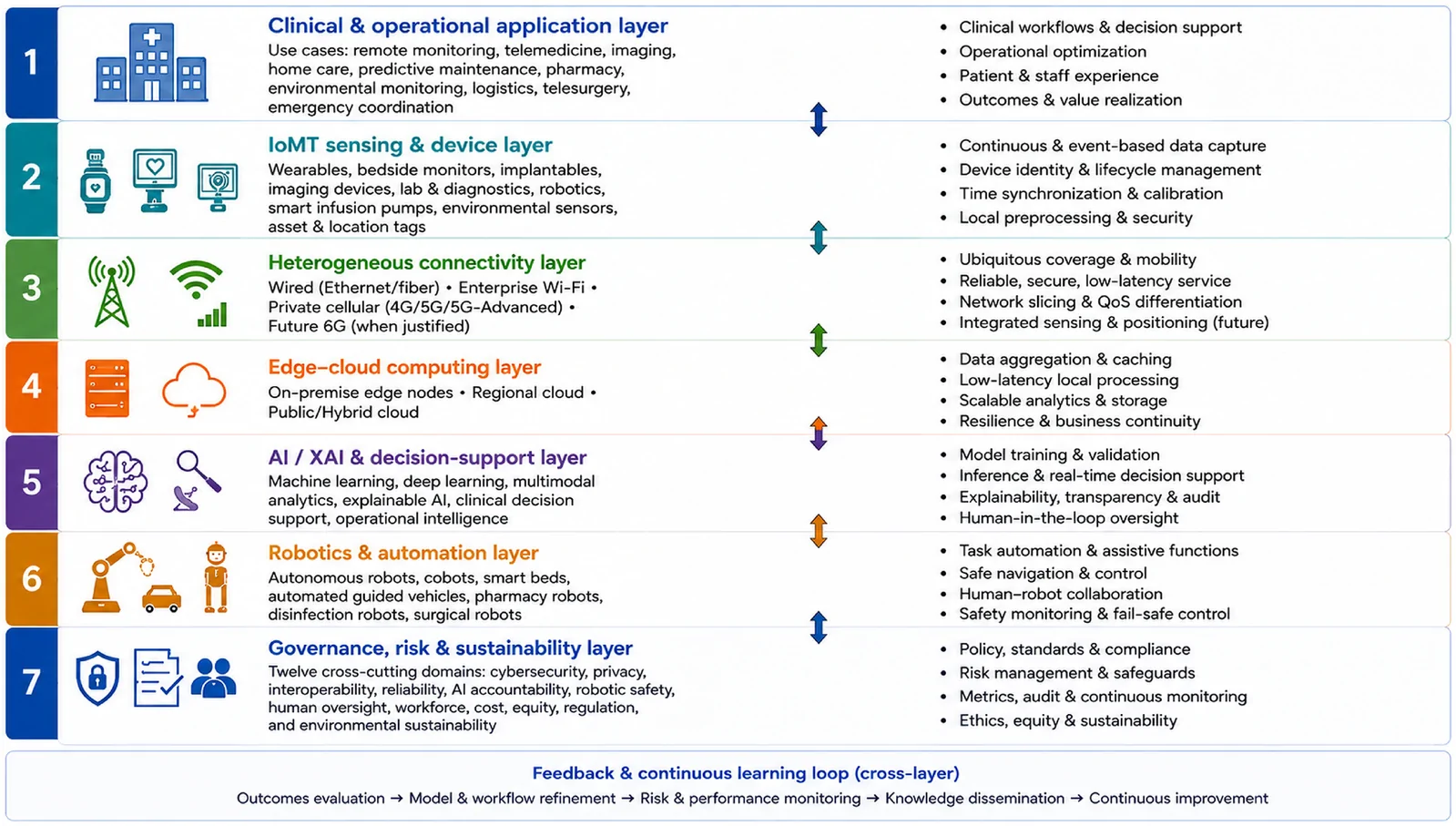
Seven-layer architecture of a prospective 6G-enabled learning hospital. The architecture integrates clinical and operational applications, IoMT sensing, heterogeneous connectivity, edge-cloud computing, AI/XAI decision support, robotics and automation, and cross-cutting governance within a continuous learning loop.

**Figure 2:**
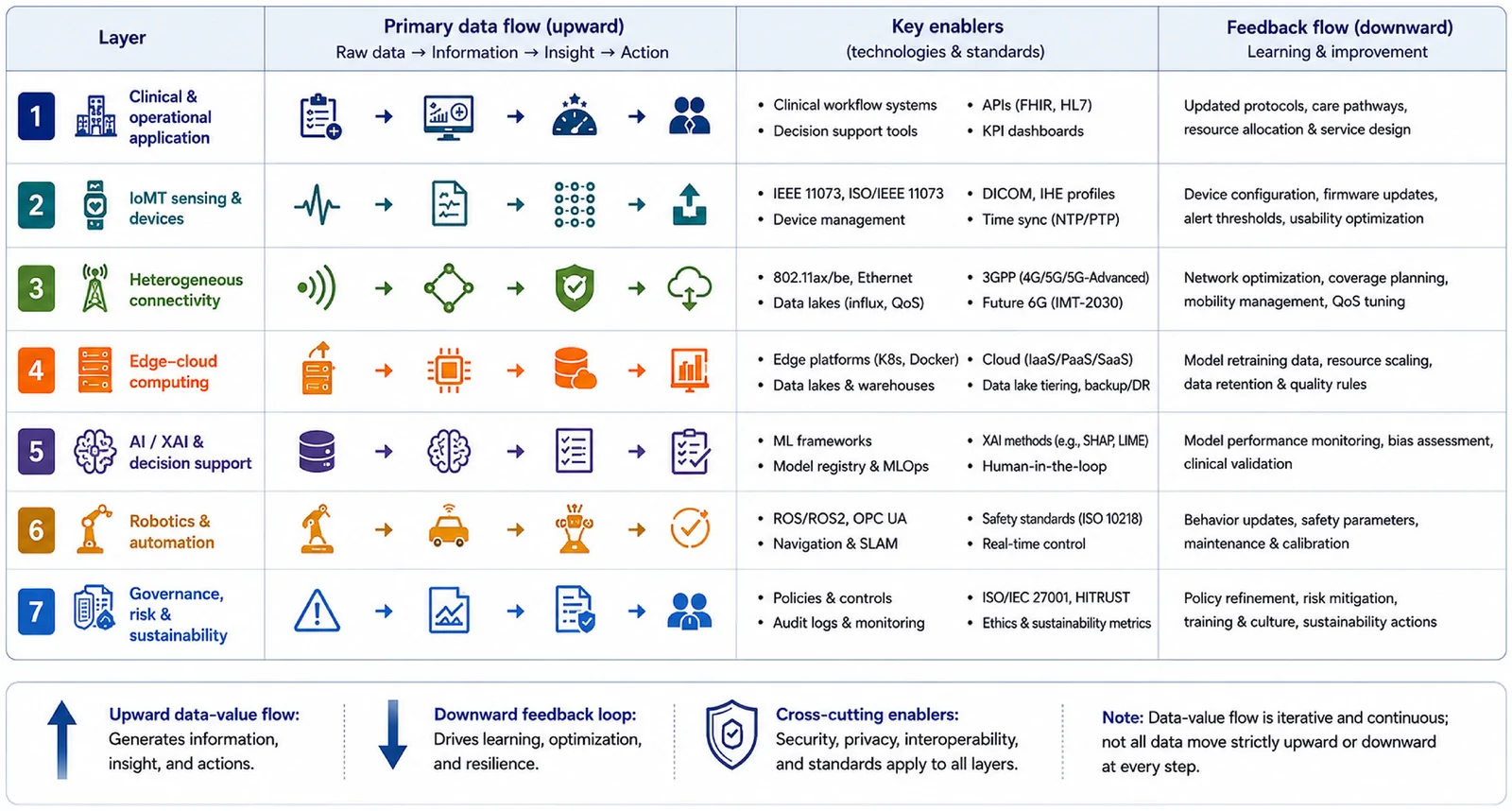
Interoperability and data-value flow across the seven-layer architecture. Data move iteratively from sensing to information, insight, and action, while feedback supports learning, optimization, and resilience across all layers.

#### 3.2.1 Clinical and operational application layer

The first layer anchors the architecture in a defined clinical or operational problem. Hospital technology should not begin with the assumption that an available 6G capability must be used. Many routine administrative, outpatient, monitoring, imaging, and operational functions can already be supported through existing networks and computing infrastructure.

Representative applications span critical-care monitoring, emergency coordination, diagnostic imaging, robotic teleoperation, chronic and home-based care, logistics and pharmacy automation, predictive maintenance, environmental safety, and digital twins or command centers. Their requirements differ substantially in timing, mobility, bandwidth, automation, safety consequences, and evidence maturity. This variation makes scenario definition a prerequisite for infrastructure selection; the comparative analysis is developed in Section 3.3.

#### 3.2.2 IoMT sensing and device layer

The IoMT layer converts physiological, diagnostic, environmental, equipment, and workflow states into computable information. It includes wearable and bedside sensors, connected imaging and laboratory devices, infusion systems, smart beds, environmental sensors, asset-tracking technologies, robotic sensors, and facility-management systems (20, 21, 23).

The principal challenge is not simply connecting more devices. Data must be reliable enough to support downstream interpretation: device identity, calibration, timestamp integrity, metadata, semantic interoperability, data minimization, and lifecycle management are therefore fundamental design requirements (21, 23, 48). Device heterogeneity also creates cybersecurity and maintenance challenges, particularly when legacy equipment and products from multiple vendors coexist.

Recent work on XAI-driven IoMT fusion further illustrates that sensing and interpretation cannot be separated conceptually. Reviews of healthcare XAI have shown increasing convergence between heterogeneous IoMT data, multimodal analytics, and interpretable decision support (35). This supports treating the sensing layer as the front end of a governed clinical-intelligence pathway rather than as an independent collection of connected devices. It does not, however, establish that these systems require 6G or improve outcomes solely because additional data are collected.

#### 3.2.3 Heterogeneous connectivity layer: current infrastructure and prospective 6G

The connectivity layer is deliberately heterogeneous. Wired networks, enterprise Wi-Fi, private cellular systems, 5G/5G-Advanced, and future 6G are likely to coexist because hospital applications differ in coverage, mobility, reliability, bandwidth, device density, and clinical criticality. Future 6G should therefore be considered one possible component of an integrated connectivity environment rather than a universal replacement for current networks.

This distinction is reinforced by the maturity of current standards. The ITU IMT-2030 framework defines prospective future communication scenarios and performance directions, including highly reliable low-latency communication, massive communication, ubiquitous connectivity, AI-assisted communication, and integrated sensing and communication (9, 10). However, these are technology-development and evaluation objectives rather than guarantees of hospital performance. At the time considered in this review, 5G-Advanced is represented principally by 3GPP Releases 18 and 19, while Release 20 includes substantial 6G study activity and normative 6G specification work is associated with Release 21 (16, 57, 58). Healthcare-oriented 6G publications remain dominated by reviews, architectures, perspectives, and engineering studies rather than mature clinical deployment (60–63).

The current baseline is also more capable than a simple 5G-vs.-6G comparison implies. 5G/5G-Advanced already incorporates quality-of-service differentiation, network slicing, mobility management, IoT support, positioning, and integration with edge computing (57, 58). Healthcare studies have demonstrated 5G-supported mobile ultrasound and emergency telemedicine, as well as selected robotic telesurgery (7, 8, 59). These studies are limited and do not establish universal clinical readiness, but they show that several applications frequently associated with 6G are already possible without it.

Clinical evaluation must therefore focus on end-to-end service, not nominal radio-interface specifications. Delay experienced by a healthcare application includes access and transport networks, encoding and decoding, application software, edge or cloud processing, sensing, device response, and—in many workflows—human action. The reported performance of 5G telesurgery illustrates this point: end-to-end delay can be substantially greater than a radio-interface latency target while still supporting selected procedures (8, 59). Consequently, a sub-millisecond future radio target should not be interpreted automatically as sub-millisecond clinical-system performance or evidence of improved safety.

A credible future 6G rationale emerges when several demanding requirements occur simultaneously and remain inadequately served after current infrastructure has been optimized. Examples include high densities of mobile medical devices; synchronized imaging, video, physiological, sensor, or haptic streams; highly reliable communication during mobility; integrated sensing and positioning; and dynamic orchestration between communication and distributed computing (9–19, 60–63). Even then, 6G is an enabling component rather than the intervention itself.

Two terminology issues require particular caution. First, network slicing is not unique to 6G; it is already part of the 5G development pathway (57, 58). Future systems may extend this concept through more adaptive and compute-aware service orchestration, but the clinically relevant issue is whether a defined application receives verifiable end-to-end service. Second, communication above 100 GHz remains an area of technical feasibility research involving propagation, channel behaviour, antenna design, and potential architectures (64). Such higher-frequency links should not be described as established hospital infrastructure.

Connectivity decisions should therefore be application-specific. High-risk functions may require bounded delay, availability targets, redundancy, continuous observability, edge fallback, graceful degradation, and predefined recovery behaviour, whereas lower-risk applications can often tolerate delayed synchronization or store-and-forward operation. In practical terms, applications can be viewed as currently deployable, transitional, or prospective high-complexity depending on whether optimized contemporary infrastructure already satisfies their requirements.

The implementation principle is straightforward: hospitals should establish measurable end-to-end requirements, benchmark them against existing wired, Wi-Fi, private 5G, 5G-Advanced, and edge architectures, and identify the residual capability gap before claiming a need for future 6G.

#### 3.2.4 Edge-cloud computing layer

The edge-cloud layer determines where data are processed, stored, and acted upon. This is a clinical and resilience decision rather than simply an information-technology design choice. Tasks differ in latency sensitivity, privacy risk, computational demand, persistence, and tolerance of network disruption (24–26).

Latency-sensitive or safety-relevant functions—such as selected monitoring alerts, robotic control, or emergency decision support—may benefit from local or edge processing so that essential functions remain available when external connectivity degrades. Cloud or central platforms are better suited to computationally intensive model development, longitudinal analytics, institutional forecasting, storage, and digital-twin simulation.

Hybrid architectures also support privacy and resilience. Local processing can reduce unnecessary movement of raw sensitive data, while federated and related privacy-preserving approaches can support cross-institutional model development without routine centralization of all source data (38, 39, 65, 66). These benefits depend on explicit policies for workload placement, access control, synchronization, redundancy, auditability, and recovery; otherwise, edge-cloud architectures can add complexity without adding clinical value.

#### 3.2.5 AI/XAI and decision-support layer

The fifth layer converts heterogeneous real-time and longitudinal information into predictions, alerts, recommendations, and operational insight. Applications range from diagnostic and imaging support to deterioration prediction, resource allocation, scheduling, and maintenance (27, 28). Within the proposed architecture, AI is treated as human-supervised decision support, not as an autonomous substitute for professional judgment.

Explainability is particularly relevant in high-stakes healthcare because users need to understand not only what a model predicts, but also its uncertainty, applicable context, and limitations (32–35). Recent application-level studies illustrate the range of current approaches. DeepXplainer combined predictive modelling with SHAP-based local and global explanations (67); EASE-Net incorporated case-specific explainability into MRI brain-tumour classification (68); and ensemble modelling with SHAP has been applied to tabular liver-disease prediction (69). Multimodal work such as SE-MMFusionGAN further demonstrates that explainability can be incorporated alongside imaging fusion, anomaly localization, and computational efficiency (70).

These studies demonstrate methodological progress rather than clinical maturity. Benchmark accuracy or the availability of an explanation does not establish clinical validity. This distinction is reinforced by clinically grounded sepsis studies: *post-hoc* SHAP and LIME analyses can help inspect model behaviour, but their explanations are approximations and cannot substitute for calibration, external validation, prospective evaluation, or assessment of clinical consequences (36). Related evidence also shows that more complex biomarkers or models do not necessarily outperform simpler clinical predictors (37).

AI governance must therefore extend beyond explainability. Relevant requirements include representative validation, calibration, subgroup assessment, drift monitoring, version control, auditability, workflow evaluation, and explicit responsibility when model output conflicts with professional judgment (29–31, 38, 39). The role of future connectivity is secondary to these requirements: a more advanced network cannot make an inadequately validated model clinically trustworthy.

#### 3.2.6 Robotics and automation layer

Robotics and automation translate digital information into physical or workflow actions. The layer includes surgical and telepresence robots, logistics systems, pharmacy automation, rehabilitation technologies, disinfection robots, and other hospital automation (49–53).

Connectivity requirements vary sharply by application. Many logistics, pharmacy, and environmental systems operate effectively using current local networks and edge processing. More demanding teleoperation may require predictable control, synchronized audiovisual or future haptic streams, distributed computation, redundancy, and reliable fallback; these conditions create a stronger potential rationale for future 6G but do not establish that 6G is inherently required.

Because automation can directly affect people and physical environments, safety depends on more than communication performance. High-risk systems require validated fail-safe behaviour, human override, simulation, environmental readiness, emergency procedures, cybersecurity, maintenance, staff training, and clearly assigned responsibility (49–51). Lower-risk robots can be deployed earlier, but they still require integration into hospital workflows and physical spaces.

#### 3.2.7 Governance, risk, and sustainability layer

The seventh layer is cross-cutting rather than terminal. Cybersecurity, privacy, interoperability, data quality, network reliability, AI accountability, robotic safety, human oversight, cost, equity, workforce readiness, regulation, and environmental sustainability affect multiple architectural layers simultaneously (38–56).

This is important because technical failures propagate. Poor interoperability may degrade AI inputs and increase workload; cybersecurity incidents may compromise both information integrity and clinical availability; and automation failure may involve technology, workflow, responsibility, and physical safety at the same time. Governance therefore cannot be reduced to compliance after deployment.

Evaluation likewise extends beyond network throughput or model accuracy. Hospital technologies should be judged through combinations of technical reliability, clinical or operational outcomes, patient safety, workflow impact, human factors, cybersecurity, cost, equity, resilience, and sustainability. The specific risk domains, safeguards, metrics, and decision gates are operationalized in Section 3.4 and Table 3 rather than repeated within the architecture itself.

Taken together, the seven layers describe a recursive pathway from clinical need to sensing, connectivity, computation, interpretation, action, and learning. Their value lies in the interfaces between layers: a technically strong component cannot compensate for poor data, an unsuitable workflow, weak validation, or absent governance. Section 3.3 therefore moves from architectural components to the comparative evaluation of the nine representative hospital use cases.

### 3.3 Clinical use cases, residual capability gaps, and deployment priorities

The seven-layer architecture provides a common structure for hospital digital transformation, but the relevance of future 6G differs substantially across clinical and operational applications. The key question is not whether a use case can be associated conceptually with 6G, but whether a clinically or operationally meaningful limitation remains after existing infrastructure has been optimized.

Accordingly, the nine representative use cases were assessed along five linked dimensions: application-level evidence maturity, capabilities already supported by current infrastructure, the nature of any residual capability gap, clinical or operational risk, and organizational readiness. Evidence supporting the healthcare application itself was considered separately from evidence supporting an incremental contribution from 6G. Table 2 summarizes the detailed evidence maturity, residual capability requirements, governance safeguards, evaluation endpoints, and deployment logic; the text below focuses on comparative interpretation rather than repeating the matrix.

**Table 2:** Clinical use-case capability, evidence-maturity, and deployment-priority matrix for prospective 6G-enabled learning hospitals.

Use case	Primary value	Current capability and evidence maturity	Residual capability gap and potential incremental 6G value	Governance requirements	Key evaluation endpoints	Deployment priority and decision logic
Remote critical care and continuous monitoring	Earlier deterioration recognition, safer escalation, continuity of high-risk monitoring	Many functions are supported by wired, Wi-Fi, private cellular, 5G, and local edge systems. Application evidence is approximately EM1–EM2. Sepsis/XAI studies strengthen the AI layer but not 6G evidence (36, 37).	Dense mobile devices, multimodal streams, edge AI, bounded delay, redundancy, and local fallback may create a residual gap. Future 6G may add mobility continuity, service differentiation, and communication-computing orchestration. 6G-specific evidence: EM4.	High: device authentication, interoperability, alarm governance, cybersecurity, AI validation and calibration, subgroup monitoring, drift, human oversight, fallback.	Latency, completeness, synchronization, availability, failover, model calibration, alert burden, response time, workload, ICU transfer, adverse events.	Intermediate for advanced monitoring. Routine monitoring should use mature infrastructure; advance only after data integration, validation, and a demonstrated connectivity gap.
Emergency care coordination	Earlier triage and pathway activation, faster specialist input, prehospital-hospital coordination	Prehospital telemedicine and selected 5G applications have EM1 field or clinical feasibility (7, 85).	Residual gaps concern mobility, coverage transitions, synchronized multimodal data, prioritization, cross-institutional interoperability, redundancy, and failover. Future 6G may add adaptive service assurance and integrated sensing/positioning. 6G-specific evidence: EM4.	High: mobile security, patient matching, emergency consent, interoperability, AI-triage accountability, fallback, clinical responsibility.	Connection time, latency distribution, interruptions, failover, data completeness, consultation success, triage concordance, pathway time, clinical outcomes, rural-urban performance.	Intermediate. Optimize 5G/private networks and interoperability first; advanced deployment requires proof that residual mobile or cross-institutional limits affect care.
Medical imaging and remote diagnostics	Faster specialist access, reduced diagnostic delay, improved multimodal interpretation	Conventional image exchange and teleradiology have EM1–EM2 foundations. Advanced XAI/multimodal imaging has EM3 benchmark evidence (68, 70).	Urgent mobile imaging, synchronized multimodal data, intraoperative guidance, sustained throughput, low jitter, mobility, and edge processing may create a gap. Future 6G may add value where current systems are insufficient. 6G-specific evidence: EM4.	Moderate-high: diagnostic accountability, image security, agreements, scanner/domain robustness, validation, calibration, uncertainty, XAI usability, professional oversight.	Transfer time, throughput, failures, edge time, image quality, accuracy, calibration, robustness, turnaround, concordance, specialist access, treatment decision, patient outcomes.	Early for conventional consultation; intermediate for advanced real-time or multimodal applications.
Robotic surgery and teleoperation	Precision intervention and remote specialist access	Selected 5G-supported telesurgery has EM1 clinical-feasibility evidence (8, 59, 90, 91).	Residual gaps include predictable end-to-end latency, jitter/packet loss, multimodal or haptic synchronization, redundant control, failover, and multi-site collaboration. 6G-specific evidence: EM4.	Very high: credentialing, patient-side emergency capability, redundancy, safe stop, local takeover/conversion, simulation, cybersecurity, consent, liability, regulation.	Latency distribution, jitter, packet loss, failover, control accuracy, synchronization, conversion, operative outcomes, workload, access, cost.	Advanced/high-risk. Future 6G-dependent teleoperation should remain in controlled research until incremental safety or access benefit is demonstrated.
Chronic disease management and home-based care	Continuity, earlier deterioration detection, reduced travel and utilization, engagement	Selected remote-management models have EM1 trial evidence (71, 72). Most current monitoring is supported by Bluetooth, gateways, broadband, Wi-Fi, and 4G/5G.	Dense multimodal home ecosystems, low-power sensing, mobility across settings, heterogeneous terrestrial/non-terrestrial connectivity, and underserved areas may create a gap. 6G-specific evidence: EM4.	Moderate-high: consent, home privacy, data minimization, responsibility, alert response, usability, literacy, reimbursement, AI validation, accessibility, affordability, equity.	Disease control, quality of life, deterioration, admissions/readmissions, adherence, data completeness, alert accuracy, workload, burden, access equity, cost-effectiveness.	Early for established models using mature connectivity; intermediate for dense multimodal systems.
Hospital logistics, pharmacy automation, and facility management	Efficiency, medication safety, workload reduction, continuity, resource optimization	Logistics robots, pharmacy automation, and smart-facility platforms have EM1–EM2 real-world evidence (73–76).	Very large robot fleets, precise positioning, dense tracking, multi-building mobility, coordinated access systems, and command environments may create a gap. 6G-specific evidence: EM4.	Moderate, higher for medication and critical facilities: chain of custody, medication safety, human-robot interaction, OT cybersecurity, fallback, vendor governance, training.	Delivery time, completion, errors, stockouts, near misses, workload, service availability, energy, faults, operating cost, total ownership.	Early/high-value. Preferred pilot domains using current connectivity; consider 6G only if scaling reveals mobility, density, localization, or coordination bottlenecks.
Predictive maintenance and service continuity	Reduced failure and downtime, improved resilience, fewer service disruptions	IoT/ML device studies provide EM2–EM3, while real-world maintenance implementations increasingly provide EM1–EM2 evidence (77–80).	Hospital-wide or multi-campus monitoring of thousands of mobile assets, dense sensing, localization, low-power operation, and distributed analytics may create a gap. 6G-specific evidence: EM4.	Moderate: asset identity, provenance, record standardization, calibration and drift, false-alert governance, engineering oversight, vendor access, cybersecurity, backup planning.	Failure, recurrence, MTBF, MTTR, downtime, availability, alert-to-maintenance time, false alerts, lead time, cost, delays, service interruption, waiting.	Early/high-value. Establish reliable data and demonstrate reductions in downtime or disruption before considering advanced connectivity.
Infection-control automation and environmental safety	Earlier environmental-risk detection, improved disinfection and compliance, safer environments	UV-C robots and environmental sensing have EM1–EM2 evidence for operational and environmental endpoints; patient-level infection evidence is less mature (81–84).	Hospital-wide dense sensing, robot coordination, localization, continuous environmental intelligence, and adaptive control may create a gap. 6G-specific evidence: EM4.	Moderate: infection-control protocols, calibration, UV-C safety, interpretation, privacy for location/behaviour monitoring, robot safety, cybersecurity, local control.	Microbial burden, ventilation/CO2, compliance, response, disinfection completion, intervention, safety, process adherence, infection outcomes, energy, cost.	Early for monitoring and selected automation. Surrogate improvements must not be interpreted as reduced infection without clinical evidence.
Digital twin and hospital command center	Situational awareness, forecasting, patient-flow coordination, resource allocation, resilience	Command centers have EM1–EM2 implementation evidence with mixed outcomes (86, 87). Hospital digital twins have EM2–EM3 pilot/modelling evidence (88, 89).	Continuous high-density synchronization of mobile IoMT, assets, patients, staff, robots, building systems, and distributed computing may create a gap. 6G-specific evidence: EM4.	High: data quality and lineage, semantic interoperability, model fidelity, calibration and uncertainty, human authority, fairness, cybersecurity, redundancy, leadership accountability.	Data completeness and latency, synchronization, forecast accuracy, calibration, model fidelity, waiting, bed flow, capacity, utilization, action/override rates, safety, resilience, cost, equity.	Intermediate-advanced. Requires mature data governance and interoperability; introduce 6G only if a validated model is demonstrably limited by current synchronization capability.

EMC indicates proximity to real-world implementation and is not a formal certainty-of-evidence grade. Across all nine use cases, application-level evidence is more mature than evidence of an incremental benefit specifically attributable to 6G. Priority reflects value, evidence maturity, residual capability gap, governance risk, and organizational readiness.

EM1, demonstrated clinical or operational feasibility; EM2, clinically grounded empirical evidence; EM3, technical, benchmark, simulation, or prototype evidence; EM4, prospective, standards-defined, or conceptual evidence.

#### 3.3.1 Applications for which current infrastructure is generally sufficient

Several high-value applications can already be implemented using wired networks, enterprise Wi-Fi, private cellular systems, 4G/5G, local IoT, and conventional edge-cloud architectures. These include chronic disease and home-based care, hospital logistics and pharmacy automation, predictive maintenance, and infection-control or environmental monitoring. Their application-level evidence is substantially more mature than evidence for 6G-specific benefit.

Remote chronic-disease management provides the clearest example. Randomized and clinical studies support structured remote care in selected conditions such as heart failure and COPD (71, 72), but the benefits arise from the complete pathway—sensing, clinical review, escalation, patient engagement, and treatment adjustment—rather than from a specific network generation. Most current home monitoring can be supported through broadband, Wi-Fi, Bluetooth, smartphones, gateways, and 4G/5G. Future 6G becomes relevant only if more distributed multimodal care creates unresolved requirements in coverage, mobility continuity, low-power device density, or cross-setting coordination.

Hospital logistics, pharmacy automation, and facility management likewise have real-world implementation evidence (73–76). Current local networks, IoT, RFID, private cellular infrastructure, and edge computing support most present applications; larger robot fleets, high-density tracking, multi-building mobility, or more precise indoor positioning may eventually expose residual capability gaps. Predictive maintenance follows the same pattern: IoT and machine-learning approaches have progressed from device-level proof of concept toward integrated maintenance workflows and hospital deployment (77–80), while the more immediate bottlenecks are often incomplete asset records, inconsistent coding, limited interoperability, and weak integration of predictions into engineering decisions.

Environmental sensing and infection-control automation also operate largely on existing infrastructure. UV-C robotic disinfection and environmental monitoring can improve selected intermediate outcomes (81–84), although reduced environmental contamination does not automatically establish reduced healthcare-associated infection (82). A future 6G role would arise only if these systems expand toward hospital-wide dense sensing, integrated localization, coordinated mobile automation, and continuous environmental intelligence. Across these four domains, high hospital value does not imply high 6G dependence; they are attractive early implementation areas precisely because meaningful outcomes can be generated using mature infrastructure.

#### 3.3.2 Applications with selective residual capability gaps

A second group comprises applications whose fundamental functions are already feasible but in which more demanding configurations may expose residual limitations in mobility, synchronization, reliability, or distributed computing. This group includes remote critical care, emergency coordination, advanced medical imaging, and digital twin or command-center systems.

Critical-care monitoring already relies on dense physiological, therapeutic, and workflow data and can use existing wired, wireless, and edge systems. Recent sepsis studies show both the potential and limitations of adding AI: explainability can assist interpretation but does not establish validity, and more complex biomarker models do not necessarily outperform simpler clinical predictors (36, 37). A future connectivity gap is therefore most plausible when large numbers of mobile devices and multimodal streams must be synchronized with predictable availability, bounded delay, redundancy, and local fallback.

Emergency care is more mobility-dependent. 5G field evidence supports ambulance-to-hospital audiovisual and mobile-ultrasound transmission (7), whereas real-world stroke telemedicine demonstrates that unstable connectivity can still disrupt prehospital care (85). Future 6G could add value if present systems cannot provide stable service across mobility, coverage transitions, distributed edge resources, and multiple synchronized streams. Imaging follows a similar pattern: routine PACS exchange and teleradiology are already mature, whereas intraoperative, mobile, or real-time multimodal imaging may require sustained throughput, synchronization, and edge processing that expose a more specific residual gap. AI imaging studies such as EASE-Net and SE-MMFusionGAN demonstrate methodological progress but do not establish 6G necessity (68, 70).

Digital twins and hospital command centers represent a system-level version of the same issue. Existing command centers have been implemented with conventional infrastructure and show mixed effects on patient flow and safety (86, 87); digital-twin platforms have reached pilot and modelling stages (88, 89). Their principal constraints remain data quality, interoperability, model fidelity, synchronization, and the institution's capacity to act. Future 6G becomes relevant only if continuously synchronized, high-density hospital representations are demonstrably limited by current communication and computing architecture. Across this group, the potential role of future 6G is selective rather than intrinsic.

#### 3.3.3 Advanced high-risk application: robotic surgery and teleoperation

Robotic surgery and remote teleoperation warrant separate treatment because they combine high clinical consequence with strict system-level performance requirements. Selected telesurgical procedures have already demonstrated feasibility using 5G, including remote urological procedures, long-distance radical cystectomy, prospective cholecystectomy, and remote prostatectomy (8, 59, 90, 91). These studies establish that telesurgery does not inherently require 6G, although they remain feasibility evidence from selected teams, patients, platforms, and network conditions rather than proof of routine readiness.

The residual capability gap is narrower but clinically important. Safety-critical teleoperation requires predictable end-to-end latency rather than low average latency alone, minimal disruptive jitter and packet loss, high availability, rapid degradation detection, redundant control pathways, and deterministic fail-safe behaviour. Future systems may also integrate multiple high-resolution video feeds, intraoperative imaging, augmented-reality information, physiological signals, instrument telemetry, or haptic feedback across geographically separated teams.

These requirements provide one of the strongest conceptual rationales for future 6G, but connectivity remains only one part of the safety case. Robotic-platform validation, local patient-side expertise, credentialing, cybersecurity, emergency conversion, device certification, human factors, informed consent, and regulatory responsibility remain fundamental. Selected 5G-supported telesurgery can therefore be considered EM1 clinical-feasibility evidence in controlled settings, while evidence for incremental clinical benefit specifically attributable to 6G remains EM4. Advanced robotic teleoperation should consequently remain a later-stage, high-governance application.

#### 3.3.4 Prioritizing applications for phased deployment

Deployment priority cannot be determined by 6G relevance alone. Five dimensions should be considered together: clinical or operational value, evidence maturity, residual capability gap, governance risk, and organizational readiness. Technology should be linked to a defined value pathway; evidence requirements should increase with risk; optimized current infrastructure should form the comparison baseline; governance intensity should reflect the consequence and reversibility of failure; and local data, workforce, cybersecurity, procurement, and evaluation capacity should be considered before scaling.

Applying these dimensions produces three broad groups. Early evidence-generating applications include logistics and pharmacy automation, predictive maintenance, environmental monitoring, selected infection-control applications, conventional remote imaging, and established home monitoring, generally using current infrastructure. Intermediate applications include advanced AI-supported monitoring, emergency coordination, multimodal or real-time imaging, integrated chronic-care systems, command centers, and digital twins. Advanced high-risk applications, represented most clearly by complex robotic teleoperation, require the strongest validation and governance before broader scaling.

These categories are not fixed: risk and capability requirements change with use-case configuration, institutional context, scale, and regulation. Table 2 therefore serves as the detailed application-level decision matrix, while Figure 3 summarizes the nine use cases qualitatively according to residual capability gap or potential incremental value of future 6G after optimization of existing infrastructure, and clinical or operational risk or governance intensity. The map is not a numerical ranking or deployment-priority score.

**Figure 3:**
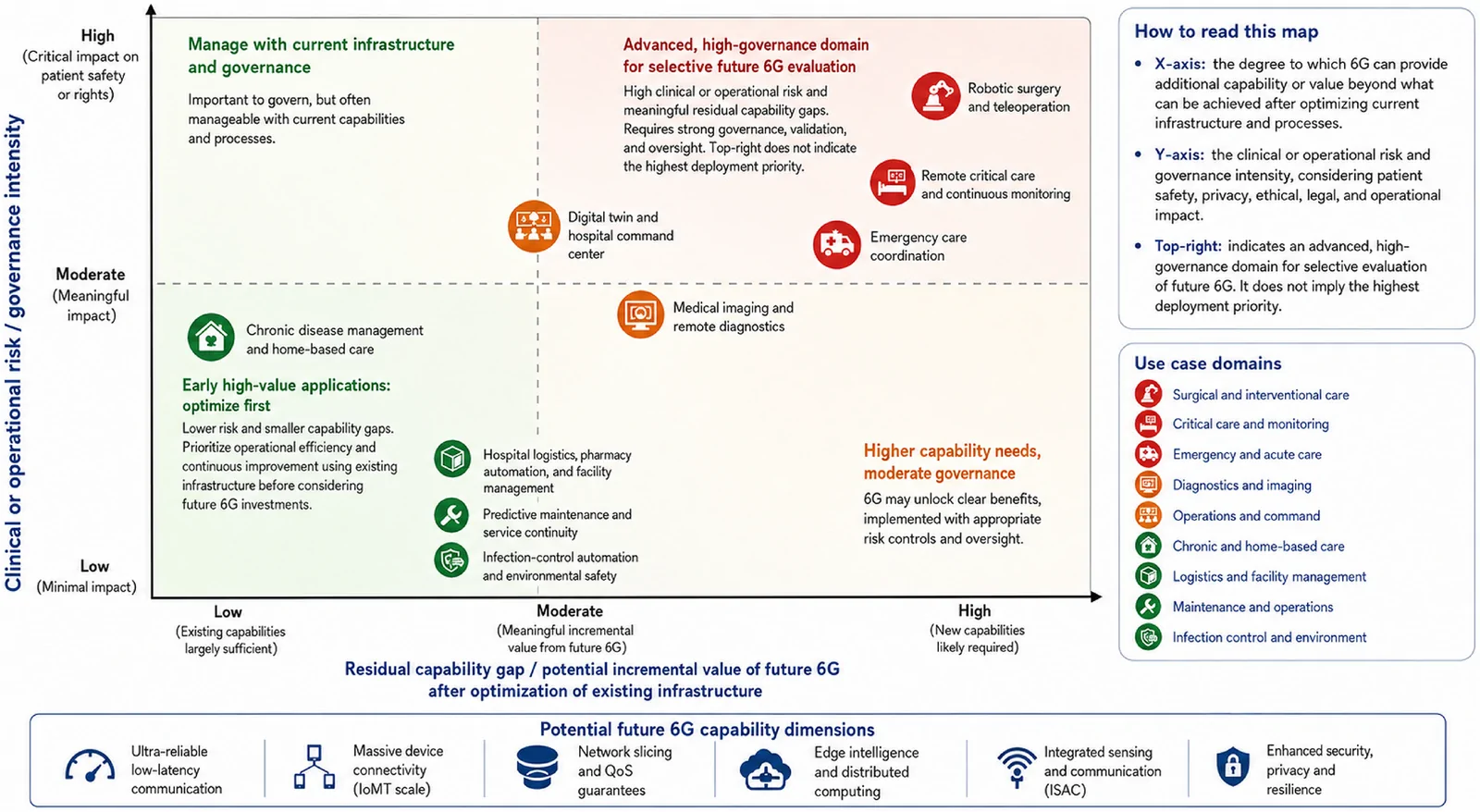
Capability-gap and governance mapping of representative hospital use cases. Applications are positioned qualitatively according to residual capability gap and potential incremental value of future 6G after optimization of existing infrastructure, and clinical or operational risk and governance intensity. Positions are heuristic rather than validated numerical scores and may vary by institutional context.

The resulting principle is deliberately conservative: hospitals should begin with meaningful problems, use the least complex infrastructure capable of addressing them, generate implementation evidence, and consider future 6G only when a residual capability gap remains and its incremental value can be demonstrated.

### 3.4 Responsible deployment: governance, risk management, and decision gates

The use-case analysis demonstrates that technical feasibility and deployment readiness are distinct. Applications with similar technologies may differ substantially in clinical consequence, reversibility of failure, data sensitivity, degree of automation, organizational complexity, and available evidence. Governance should therefore be proportional to the consequences of failure rather than to technological novelty itself.

The framework treats governance as an embedded property of the learning hospital rather than a final compliance layer. Risks are intentionally non-mutually exclusive: poor data quality may simultaneously affect AI validity, interoperability, equity, and clinical safety, while compromise of a connected robotic system may involve cybersecurity, network reliability, physical safety, and organizational accountability. Readiness should therefore be assessed through relevant risk domains and explicit decision gates rather than a compensatory numerical score. Table 3 operationalizes this principle across twelve governance domains; the text below emphasizes how these domains function together rather than repeating the safeguards and metrics listed in the table.

**Table 3:** Risk-governance-metric framework for responsible deployment of learning-hospital technologies.

Governance domain	When the risk becomes relevant	Key safeguards	Evaluation focus	Decision gate before scaling
Cybersecurity and operational resilience	Connected devices, AI platforms, robots, IoMT, remote access, or operational technologies create new attack surfaces or service dependencies	Identity management; segmentation; secure updates; access control; incident response; redundancy and recovery	Security incidents; vulnerability status; downtime; recovery; audit findings	Controls tested; critical failure scenarios addressed; recovery validated
Privacy and data protection	Continuous sensing, home monitoring, location or behavioural data, or secondary data use involve sensitive information	Data minimization; purpose limitation; consent/governance; access control; retention	Unauthorized access; privacy incidents; compliance; patient/staff understanding	Data governance approved; privacy risk acceptable
Interoperability and legacy-system integration	Multiple devices, institutions, vendors, or systems must exchange data	Standardized models; interface validation; device identity; semantic mapping; integration testing	Completeness; exchange reliability; integration errors; reconciliation burden	Required systems integrated; data flow validated
Data quality and clinical validity	AI, digital twins, predictive models, or decision support depend on heterogeneous data	Provenance; quality monitoring; timestamp consistency; validation; uncertainty assessment	Missing data; error rates; input quality; reliability of derived outputs	Quality sufficient for intended use; limitations documented
Network reliability and clinical safety	Applications depend on timely communication for monitoring, coordination, or control	Service requirements; redundancy; failover; edge fallback; performance monitoring	Latency distribution; jitter; packet loss; availability; recovery	Performance demonstrated under realistic conditions
AI accountability and explainability	AI influences diagnosis, prediction, prioritization, or operational decisions	External validation; calibration; bias assessment; explainability; human oversight; monitoring	Accuracy; calibration; subgroup performance; drift; explanation usefulness; clinical impact	Performance and limitations acceptable for intended use
Robotics and automation safety	Systems perform physical actions or alter workflows	Human override; safe stop; simulation; environmental validation; maintenance; incident reporting	Failure events; override; workflow disruption; safety events	Safety procedures validated; responsibility established
Cost and procurement sustainability	Technologies require major investment, vendor dependence, or long-term maintenance	Lifecycle costing; interoperability requirements; procurement governance; vendor assessment	Total cost; maintenance; upgrade feasibility; resource use	Sustainable financing and lifecycle management established
Equity and digital inclusion	Access depends on connectivity, devices, digital skills, or socioeconomic factors	Accessibility design; alternative pathways; support; subgroup evaluation	Access differences; participation; retention; outcome disparities	No unacceptable inequity identified
Environmental sustainability	Large-scale sensing, computing, devices, or automation increase resource consumption	Energy-aware design; lifecycle assessment; efficient computing; responsible disposal	Energy use; equipment lifecycle; carbon impact; waste	Environmental burden acceptable relative to benefit
Workforce readiness and organizational adoption	Technologies change professional roles, workflows, or decision processes	Training; workflow redesign; user involvement; support structures	Adoption; workload; acceptance; training; workarounds	Staff capability and organizational readiness demonstrated
Regulatory, ethical, and organizational accountability	Technologies influence clinical decisions, autonomy, responsibility, or patient rights	Governance ownership; regulatory review; incident review; accountability; continuous monitoring	Compliance; incidents; accountability clarity; ethical concerns	Responsibility structure established; ongoing oversight available

Governance domains are intentionally cross-cutting and non-mutually exclusive. A single application may activate multiple domains simultaneously. Scaling should occur only when all materially relevant domains have acceptable readiness, measurable benefits have been demonstrated, and foreseeable failure modes have been identified and controlled. The framework is informed by AI governance, medical-device cybersecurity, socio-technical implementation, and digital-health guidance (38, 46, 54, 55).

#### 3.4.1 Four cross-cutting governance requirements

Digital trust and information integrity provide the foundation for every connected application. Cybersecurity should be treated as a patient-safety and service-continuity issue because medical devices, IoMT platforms, edge nodes, operational technologies, and external services increase both the hospital attack surface and dependence on digital infrastructure (41–47). Increasing availability of physiological, imaging, behavioural, location, and operational data also requires data minimization, purpose limitation, access governance, appropriate retention, interoperability, provenance, semantic consistency, timestamp integrity, and interface validation (38–40, 45, 48).

Safety-critical technical assurance becomes more stringent when digital systems influence time-sensitive decisions or physical actions. Network performance should be evaluated through end-to-end availability, delay distribution, jitter, packet loss, failover, and recovery rather than peak throughput or nominal radio latency. AI requires calibration, validation, subgroup assessment, drift monitoring, workflow evaluation, and human oversight even when explanations are available (29–40, 92). Robotics adds physical-system risks and therefore requires safe-stop behaviour, manual or local override, simulation, maintenance, cybersecurity, and clearly defined responsibility (49–51).

Sustainable and equitable implementation determines whether technical feasibility can produce durable hospital value. Total cost includes integration, licensing, cybersecurity, maintenance, workforce training, energy use, vendor dependence, and replacement cycles (54–56). Equity should be evaluated empirically through access, affordability, accessibility, digital literacy, participation, and outcome differences (39, 56), while environmental evaluation should account for energy demand, hardware lifecycle, electronic waste, and computational burden.

Organizational and institutional accountability connects these safeguards to practice. Hospitals require trained users, workable pathways, clear decision rights, technical support, incident-review processes, and responsibility for approving, monitoring, modifying, or withdrawing technologies (54, 55). These four requirements form a cross-cutting governance architecture rather than independent silos; the twelve operational domains and corresponding safeguards, metrics, and scaling conditions are summarized in Table 3.

#### 3.4.2 Decision gates for deployment and scaling

Responsible implementation is organized through four sequential decision gates. Gate 1—problem and value definition—asks whether the application addresses a clear problem, whether expected benefit is measurable, and whether reasonable current alternatives have been assessed. Gate 2—readiness assessment—determines whether the available evidence is appropriate for the application's risk and whether data quality, interoperability, cybersecurity, workforce preparation, accountability, fallback procedures, and evaluation methods are sufficiently mature to support a pilot.

Gate 3—pilot evaluation—asks whether the application produces its intended clinical or operational value under realistic conditions. Technical performance should be interpreted together with safety, workflow impact, user behaviour, cost, equity, cybersecurity, and unintended consequences. Gate 4—scale-up and continuous learning—requires reproducible value, acceptable residual risk, organizational capacity for long-term operation, and continued oversight. Post-deployment surveillance should support four responses: continue, modify, pause, or withdraw.

Prospective 6G deployment adds one further condition within these gates: a meaningful residual capability gap must remain after reasonable optimization of existing wired, Wi-Fi, private cellular, 5G/5G-Advanced, IoT, and edge-cloud infrastructure. A technically advanced network should not advance an application through the governance pathway if the application itself lacks adequate evidence, the hospital is not ready to operate it safely, or the expected incremental value has not been demonstrated.

### 3.5 Implementation roadmap: from evidence generation to selective 6G scaling

The implementation roadmap translates the preceding architecture, use-case analysis, and governance framework into a staged hospital strategy. It should not be interpreted as a sequence of network upgrades. Many applications can already generate meaningful value using current infrastructure, whereas evidence of an incremental clinical or operational benefit specifically attributable to 6G remains limited. Progression should therefore depend on evidence, residual capability gaps, and governance gates rather than a predetermined technology timetable.

Figure 4 summarizes the five phases, four decision gates, continuous reassessment, and the EMC continuum. The text below focuses on the purpose of each phase and the condition for progression rather than reproducing the operational details shown in the figure. The roadmap is intentionally reversible: hospitals may remain within, return to, or repeat a phase when evidence or local conditions change.

**Figure 4:**
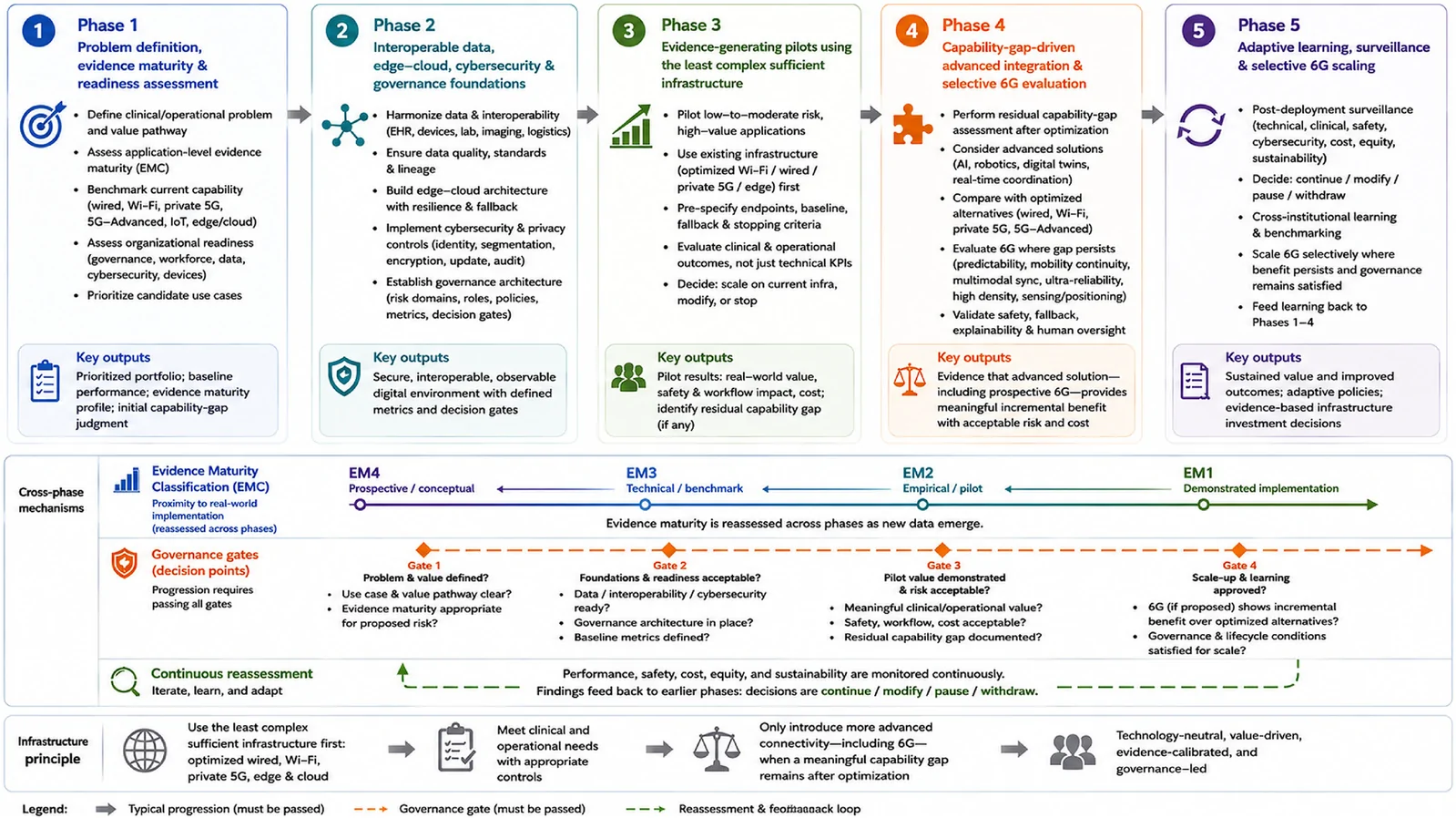
Implementation roadmap from evidence generation to selective 6G scaling. Five implementation phases are supported by four governance decision gates, continuous reassessment, and an evidence-maturity continuum from EM4 prospective evidence toward EM1 demonstrated implementation. 6G evaluation begins only when a meaningful residual capability gap remains after optimization of current infrastructure.

#### 3.5.1 Phase 1: problem definition, evidence maturity, and readiness

Implementation begins by defining a measurable clinical or operational problem and the mechanism by which the proposed intervention is expected to improve it. Candidate applications should then be assessed according to evidence maturity, baseline performance, clinical or operational risk, and local readiness. Readiness includes network and edge infrastructure, data quality, interoperability, cybersecurity, device inventory, workforce capability, procurement arrangements, clinical governance, and evaluation capacity (54–56). A central task is to benchmark the proposed requirement against current alternatives before assuming that advanced connectivity is required. The output of Phase 1 is a prioritized portfolio of defined problems and evidence-supported candidate applications, not a technology procurement list.

#### 3.5.2 Phase 2: data, interoperability, cybersecurity, and edge-cloud foundations

The second phase develops infrastructure that remains valuable regardless of whether 6G is ultimately adopted. Hospital information, imaging, laboratory, pharmacy, device, IoMT, maintenance, robotic, and operational systems should exchange sufficiently standardized and traceable information; data-quality controls should address missingness, inconsistency, calibration, provenance, and timestamp integrity (45, 48). Computing architecture should reflect clinical requirements, with local or edge processing for selected time-sensitive or safety-relevant functions and larger central or cloud resources for longitudinal analytics, model development, forecasting, and simulation (24–26). Cybersecurity, privacy, vendor access, device identity, recovery procedures, and activated governance domains should be defined before reliance on connected systems increases (38–48).

#### 3.5.3 Phase 3: evidence-generating pilots using sufficient current infrastructure

Phase 3 tests whether an application creates real value rather than merely demonstrating technical operation. Early pilots should favour measurable, manageable-risk applications such as predictive maintenance, logistics or selected pharmacy automation, environmental monitoring, conventional remote imaging, established home-care models, and selected monitoring functions. They should begin with the least complex infrastructure capable of meeting the intended requirement, providing a contemporary comparator and preventing improvements caused by workflow redesign, integration, or edge computing from being attributed incorrectly to a new network generation. Applications demonstrating value may scale using current infrastructure; only those constrained by a documented and clinically or operationally meaningful technical limitation should proceed to advanced capability evaluation.

#### 3.5.4 Phase 4: capability-gap-driven advanced integration and selective 6G evaluation

The fourth phase addresses applications in which greater scale or complexity creates residual requirements that optimized current infrastructure cannot adequately satisfy. Relevant limitations may include mobility continuity, predictable end-to-end service, high-density device coordination, synchronization of multiple high-volume streams, integrated sensing or positioning, or large-scale orchestration of communication and distributed computing. Advanced AI, robotics, digital twins, multimodal systems, and future 6G should enter at this stage only when their role is linked to a defined gap.

Where 6G is evaluated, comparison with optimized wired, Wi-Fi, private 5G, 5G-Advanced, and edge-cloud alternatives should be explicit. This is particularly important for applications traditionally labelled as 6G use cases: selected telesurgery has already been demonstrated using engineered 5G systems (8, 59, 90, 91), while advanced imaging should justify new connectivity through a constrained real-time workflow rather than file size alone. Progression requires evidence that closing the residual capability gap produces a meaningful improvement that justifies additional risk, cost, and complexity.

#### 3.5.5 Phase 5: adaptive learning, surveillance, and selective scaling

The final phase is defined by sustained institutional learning rather than by the amount of advanced technology deployed. Clinical, operational, technical, cybersecurity, workforce, cost, equity, and sustainability indicators should become part of routine post-deployment surveillance. Performance can change as populations, workflows, staff, devices, AI models, software, cyber threats, infrastructure, and regulation evolve; continued deployment should therefore remain conditional rather than permanent. Evidence should support decisions to continue, modify, pause, or withdraw an application, while cross-institutional collaboration can support benchmarking, safety registries, interoperability standards, simulation, and shared learning.

Selective 6G scaling follows the same principle. Expansion is justified only when the application continues to demonstrate value, the residual capability gap persists at scale, and governance requirements remain satisfied. If a less complex infrastructure performs equally well, migration to 6G solely for technological uniformity is not justified. Across the five phases, hospital transformation is therefore driven by progressively stronger evidence linking clinical need, application value, residual technical requirements, and governance readiness.

## 4 Discussion

This review addressed a narrower and more consequential question than whether 6G can technically support healthcare: under what clinical, technical, organizational, and governance conditions would future 6G capabilities justify changes to hospital infrastructure and care delivery? The synthesis indicates that the relevant transition is not simply from 5G to a faster network. It is a transition from connectivity-centred digitalization toward a learning hospital in which an evidence-supported application, a demonstrable residual capability gap, and a governed feedback loop are aligned.

The principal contribution of the framework is therefore not a catalogue of future technologies. It is a decision logic for separating three issues that are often conflated: whether a healthcare application has value, whether current infrastructure can support it, and whether future 6G capabilities would add clinically or operationally meaningful performance. Once these questions are separated, a more selective deployment strategy follows. Hospitals can implement many high-value applications using existing digital, wired, Wi-Fi, edge, private cellular, and 5G infrastructures, while reserving future 6G investment for situations in which a specific capability gap remains and the application has sufficient evidence and governance readiness.

### 4.1 From connectivity-centred smart hospitals to learning hospitals

The learning-hospital concept developed in this review builds on the view that research, care delivery, and institutional improvement should become progressively integrated rather than remain separate activities (1–3). The present framework extends that reasoning to communication and computational infrastructure: sensing, connectivity, edge-cloud processing, AI, automation, and governance have value only when they form a closed loop through which observed outcomes alter subsequent decisions, workflows, models, and policies.

This interpretation places the hospital within a socio-technical rather than purely technical frame. Health-information technology performance depends on interactions among hardware, software, data, people, workflows, organizational structures, regulation, and external conditions (54). A hospital can possess advanced networks and still fail to learn. Fragmented data, weak clinical ownership, poorly designed alerts, limited interoperability, or absent decision authority can interrupt the learning cycle even when information is transmitted rapidly.

The seven-layer architecture should not be read as a linear technology stack. Its layers describe interdependent functions: clinical problems define information requirements; sensing creates observations; connectivity and computation determine whether those observations are available at the appropriate place and time; AI or other analytical methods support interpretation; automation translates selected decisions into action; and governance constrains the entire process. Feedback from outcomes then changes the system. Governance is not the seventh step after deployment, but the set of rules that determines how every layer is designed, monitored, and revised.

This framing distinguishes the present work from much of the 6G literature. Foundational papers have appropriately concentrated on future service classes, network architectures, spectrum, sensing, computing, and target performance (11–15). Healthcare-focused studies have translated these capabilities into remote monitoring, imaging, robotic procedures, and pervasive connected care (17–19). A recent scoping review has further expanded the discussion to infrastructure, ethics, security, and policy (93). These contributions establish technological plausibility and a research agenda, but they do not by themselves demonstrate that a hospital application requires 6G or that higher network performance improves care.

The distinction matters because a substantial portion of the functionality attributed to future 6G is already partially achievable. A recent operating-room analysis explicitly recognized that several envisioned functions can be supported by fixed networks, Wi-Fi, or 5G (60). Field evaluation has demonstrated 5G-supported ambulance-to-hospital audiovisual and ultrasound transmission (7), while prospective controlled evidence has reported selected telerobotic procedures over a 5G network (8). These studies do not establish universal safety or scalability, but they shift the burden of proof. The relevant question is no longer whether advanced remote care can exist before 6G; it is which residual limitations remain after current infrastructure has been optimized.

This observation changes how latency should be discussed. Clinical systems experience end-to-end delay, not radio-interface delay alone. Encoding, transport, processing, rendering, device response, clinical interpretation, and human action all contribute to whether information arrives in time to affect care. A future reduction in one component is clinically valuable only if it removes a meaningful workflow constraint. Peak throughput and nominal latency should therefore be treated as enabling parameters, not surrogate clinical outcomes.

### 4.2 Evidence maturity and the selective role of 6G

Separating application-level evidence from connectivity-specific evidence is one of the most important findings of the synthesis. The maturity of the care model, sensing technology, analytical method, automation component, and communication infrastructure can differ substantially within the same use case. A remote-care pathway can have randomized clinical evidence even though no evidence shows that 6G is needed to deliver it. Conversely, a future network architecture can be technically sophisticated while the proposed clinical application remains insufficiently validated.

The chronic-care literature illustrates this separation clearly. Structured remote patient management in heart failure has been evaluated in a large multicentre randomized trial and produced measurable benefit in a defined population (71). Those findings support the care model—continuous monitoring combined with organized clinical review and intervention—not a specific network generation. Replacing the underlying connection with 6G would require separate evidence that new infrastructure resolves an existing limitation and improves access, continuity, safety, cost, or outcomes beyond what the established pathway can achieve.

The same principle applies to AI. Recent XAI literature demonstrates rapid technical progress in combining IoMT data, machine learning, and local or global explanations (32–35). However, the evidence spans different maturity levels. Public-dataset and benchmark studies establish reproducibility, comparative algorithmic performance, and technical feasibility; they do not establish transportability or clinical benefit. Patient-level cohort studies provide stronger grounding but still require external and prospective validation.

The two sepsis studies incorporated into the revised synthesis are instructive. One showed that a multivariable model could outperform individual biomarkers for respiratory-source identification and used SHAP and LIME to examine model behaviour, while emphasizing that *post-hoc* explanation is not evidence of validity (36). The other found that a comparatively parsimonious model based on routine clinical information outperformed more complex endothelial and inflammatory biomarker models for selected outcomes (37). Together, these studies support two conclusions: explainability should complement rather than replace validation, and technological or biological complexity should not be presumed to confer greater clinical value.

The EMC is not a hierarchy in which standards evidence or engineering research is judged inferior to clinical evidence. Different evidence types answer different questions. Standards and communication research define feasible architectures and future objectives; clinical studies assess safety and usefulness; operational studies assess workflow and institutional value. Problems arise when evidence is transferred across categories—for example, when a throughput target is interpreted as proof of better outcomes, or successful AI performance is used to justify 6G connectivity.

The use-case analysis consequently supports a staged portfolio rather than a hospital-wide technology transition. Logistics, selected pharmacy automation, environmental sensing, predictive maintenance, conventional remote imaging, and established home-monitoring pathways can generate useful evidence with current infrastructure (71–84, 94). These are suitable early domains not because they are trivial, but because their value pathways are measurable and many failures are observable and reversible.

More complex monitoring, emergency coordination, multimodal imaging, command centres, and digital twins require stronger foundations in data quality, interoperability, workflow design, and governance. Their value cannot be inferred from technical integration alone. A hospital operational digital-twin platform has demonstrated proof of concept (88), but proof of concept is not evidence of improved outcomes. Likewise, a real-world command-centre evaluation found that centralized visibility did not produce consistent improvement across patient-flow and data-quality outcomes (86). Mixed evidence is especially valuable because it shows that real-time information creates an opportunity for action, not the action itself.

At the high-risk end, robotic teleoperation requires the strongest evidentiary and governance thresholds. Existing 5G studies show that selected procedures are feasible in controlled environments (8, 59, 90, 91), weakening any general claim that robotic surgery intrinsically requires 6G. Future 6G relevance instead lies in narrower residual requirements: highly predictable end-to-end performance, multimodal synchronization, advanced redundancy, distributed control, or multi-site collaboration that current systems cannot deliver reliably. Network performance remains only one part of an integrated surgical safety case.

### 4.3 Governance as an operating architecture

The risk-governance-metric framework advances a non-compensatory model of technology readiness. Composite maturity scores simplify comparison but can conceal critical weaknesses. A high-risk application should not scale because strong throughput, accuracy, or projected return on investment mathematically offsets the absence of failover, weak cybersecurity, or unclear responsibility. The decision-gate approach treats some conditions as prerequisites.

Overlap among risk domains is deliberate. Interoperability failure can degrade data quality, produce unsafe AI inputs, create workflow burden, and weaken accountability simultaneously. A cyber incident involving a robotic or monitoring platform can affect confidentiality, availability, physical safety, and service continuity. The framework organizes domains for analytical clarity without treating them as independent silos.

Interoperability remains a foundational constraint. Digital medicine depends on the ability to exchange and interpret data across systems; without that capability, AI, analytics, mobile applications, and cross-institutional care remain limited (95). Increasing transmission speed in a semantically fragmented environment can move inconsistent data faster without creating usable knowledge. Hospitals should establish authoritative sources, common identifiers, terminology governance, timestamp integrity, provenance, and interface testing before introducing more demanding real-time applications.

Cybersecurity has the same foundational role. Analysis of medical-device and healthcare-software vulnerabilities has identified weaknesses across records, infusion devices, imaging-related software, and connected platforms (44). As communication and computing become more distributed, the boundary between information security and patient safety becomes less defensible. Authentication, segmentation, update governance, vendor-access control, monitoring, incident response, and recovery should be incorporated into the clinical safety case (41–47).

Privacy-preserving architectures can reduce unnecessary concentration of sensitive information but do not remove the need for institutional governance. Federated learning offers a means of developing models across organizations without directly centralizing raw data (65, 66). Its usefulness will depend on heterogeneity, security, model ownership, audit rights, fairness, and capacity to investigate errors across institutions. Privacy technologies should be evaluated by their effect on the complete clinical and governance pathway, including computational overhead and accountability.

AI governance requires a similar distinction between model properties and clinical performance. Early-stage clinical evaluation guidance emphasizes human factors, workflow, safety, and transparent reporting (31). This is consistent with the framework's requirement that discrimination, calibration, subgroup performance, alert burden, override behaviour, and downstream outcomes be evaluated together. An interpretable model can still be poorly calibrated; an accurate model can increase workload; and a useful model in one institution can degrade after changes in population, devices, or practice.

Organizational readiness ultimately determines whether safeguards function outside formal documentation. Socio-technical and implementation frameworks show that adoption depends on interactions among the condition, technology, value proposition, users, organization, wider system, and change over time (54, 55). The revised framework translates that insight into gates. Training, decision rights, fallback, incident review, procurement, post-deployment surveillance, and authority to pause or withdraw are not secondary details; they constitute the institution's capacity to govern technology.

The learning-hospital concept also changes the meaning of failure. A pilot that is modified, paused, or withdrawn after identifying unacceptable burden or insufficient value has generated useful knowledge. Continued deployment despite harm, inequity, or non-value represents a failure to learn. The four post-deployment options—continue, modify, pause, and withdraw—are therefore essential elements of adaptive governance.

### 4.4 Implications for technology development, hospital management, and policy

For technology developers, the framework calls for clinically anchored specifications. Performance targets should derive from the workflow and failure consequences rather than maximum network capability. Studies should report complete end-to-end architectures, including sensing, encoding, transport, edge or cloud processing, application software, device response, and human interaction. Comparisons with optimized current infrastructure are essential. Without a comparator, an improvement attributed to 6G could reflect better system engineering rather than a capability unique to the network.

Developers should state which component of an application is evidence-supported and which remains prospective. A multimodal AI model, robotic platform, and future communication system can each have different maturity. Reporting them as a single 6G-enabled intervention obscures uncertainty and limits replication.

For hospital leaders, preparation for 6G should begin before procurement of 6G infrastructure. Data governance, interoperability, device inventory, cybersecurity, edge architecture, workforce capability, and outcome measurement are useful investments regardless of which future network becomes available. Institutions without these foundations risk using faster connectivity to amplify fragmentation.

Early implementation should favour use cases with measurable value and manageable risk. Predictive maintenance, logistics, selected pharmacy automation, environmental monitoring, established remote consultation, and carefully defined home monitoring can test governance and generate operational evidence (71–84, 94). The objective is not to label these pilots as 6G projects, but to build organizational capacity for more demanding applications.

Investment decisions should remain technology-neutral. Procurement should specify service characteristics, interoperability, data access, cybersecurity, auditability, lifecycle support, and exit arrangements rather than assume a network generation guarantees performance. Total cost should include integration, maintenance, security, energy, training, vendor dependence, and replacement.

For policymakers and regulators, the framework identifies a need to connect telecommunications performance with clinical risk. Generic network indicators are insufficient for high-risk care. Application-specific standards should define how latency, availability, failover, synchronization, and degradation are measured end to end and how they relate to clinical fallback. Regulatory evaluation should consider the combined system when network, software, AI, and robotic components interact (38–40, 46, 92).

Policy also has a distributive dimension. 6G-enabled services could improve regional collaboration and specialist access but infrastructure-intensive deployment could widen differences between tertiary urban hospitals and resource-constrained institutions. Equity assessment should precede scaling. Shared regional platforms, hybrid connectivity, public infrastructure investment, accessibility requirements, and non-digital alternatives are more likely to reduce disparities than market diffusion alone (39, 56).

Environmental sustainability deserves equivalent attention. Dense sensing, edge nodes, cloud workloads, network equipment, and short replacement cycles create resource costs easily omitted from feasibility studies. Energy and lifecycle measures should be reported alongside latency, accuracy, and efficiency, particularly when an application operates continuously or at hospital scale.

### 4.5 Research agenda derived from the framework

The immediate research need is comparative rather than purely demonstrative. Future studies should not ask only whether a 6G-oriented prototype works. They should compare it with an optimized wired, Wi-Fi, private 5G, 5G-Advanced, or hybrid edge-cloud alternative and identify the precise residual limitation removed. This is essential for estimating incremental value and avoiding attribution of general system improvement to 6G.

Application-specific safety thresholds are also needed. Average latency has limited meaning for a safety-critical workflow if occasional extreme delays, jitter, packet loss, or failure transitions are not characterized. Teleoperation, emergency coordination, critical-care monitoring, and real-time image guidance require different thresholds and consequences of degradation. Engineering experiments should be linked to simulation, human-factors testing, incident scenarios, and clinical endpoints. Rare but serious failures will require registries, post-market surveillance, and high-fidelity simulation in addition to conventional trials.

A second priority is linked benchmark resources. Existing datasets often isolate one layer—physiological signals, images, maintenance logs, or network measurements. Evaluation of learning hospitals requires datasets that connect technical performance with workflow events, clinician actions, outcomes, and governance indicators. For monitoring, this could include signal quality, transmission delay, model output, alert presentation, clinician response, escalation, and outcome. Such linkage would allow researchers to determine where a failure entered the pathway.

Clinical AI research should move beyond the routine combination of high discrimination and *post-hoc* explanation. The reviewed studies show that explanation tools can reveal model behaviour and support plausibility checks, but also expose instability, redundancy, threshold dependence, and overfitting concerns (35–37, 67–70). Future evaluation should include calibration, decision-curve analysis, precision-recall performance for rare outcomes, explanation stability, clinician comprehension, appropriate reliance, override behaviour, and downstream decision quality. Prospective multicentre studies are needed to determine whether explanations improve care rather than merely make outputs appear acceptable.

Privacy-preserving and cross-institutional learning is another priority. Federated approaches align with regional learning-hospital networks, but should be tested under real differences in standards, devices, populations, governance, and resources (65, 66). Research should compare federated, centralized, and local approaches in privacy risk, performance, communication burden, auditability, fairness, and institutional control. Technical guarantees should be accompanied by policies for model ownership, withdrawal, incident investigation, and patient communication.

Digital twins and command centres require stronger implementation research. Pilot platforms establish feasibility, while mixed command-centre findings show that visibility alone does not ensure improvement (86–89). Future studies should distinguish software effects from concurrent organizational redesign, use controlled or quasi-experimental methods where feasible, and report how predictions produce actions. Model fidelity, data quality, decision authority, and organizational response should be treated as co-determinants.

Finally, economic, equity, workforce, and sustainability evaluations should be embedded prospectively. Cost-effectiveness will differ markedly between predictive maintenance, monitoring, digital twins, and teleoperation. Distributional analyses should identify who gains access, who carries additional workload, and who is excluded. Longitudinal studies are needed because maintenance cost, cybersecurity burden, model drift, staff turnover, and obsolescence are unlikely to be visible during short pilots.

### 4.6 Strengths and limitations

This review has several strengths. It integrates communication engineering, digital health, clinical AI, IoMT, robotics, cybersecurity, hospital management, and implementation science within a single hospital-level argument. It avoids assuming that maturity of an application establishes maturity of 6G. The separation of current capability, residual gap, potential 6G value, evidence maturity, governance, and endpoints provides a more discriminating basis for decisions than a general list of future use cases.

The framework also introduces explicit decision gates. Rather than treat governance as broad principles, the risk-governance-metric structure links activated risks to safeguards, observable indicators, and scaling conditions. Its non-compensatory logic is particularly relevant to high-risk systems in which one unresolved weakness can invalidate otherwise strong performance.

Several limitations qualify these contributions. First, this is a narrative and conceptual synthesis rather than a systematic review. Although the revised Methods describe literature identification and evidence roles, the review did not undertake exhaustive PRISMA screening, formal risk-of-bias assessment, or certainty grading. Source selection and interpretation involved author judgment, and relevant studies may have been missed.

Second, the 6G-specific evidence base remains immature. Much of the literature consists of standards objectives, technical visions, simulations, prototypes, and prospective architectures (9–19, 57–64, 93). These sources are appropriate for possible capabilities but cannot establish clinical necessity, comparative effectiveness, cost-effectiveness, or safety. The framework relies partly on evidence from adjacent technologies—5G, edge computing, IoMT, AI, robotics, and digital twins—to infer requirements and governance needs. Such transfer supports conceptual development but is not direct validation of 6G-enabled hospitals.

Third, the EMC, prioritization, and capability-risk mapping are author-developed heuristic tools. They have not been validated as measurement instruments and should not be interpreted as formal evidence grades or universal rankings. Positioning will vary according to infrastructure, scale, clinical context, geography, and regulation. Empirical testing, stakeholder consultation, and structured consensus are required to refine the classifications and assess reliability.

Fourth, evidence maturity varies within each use case. Medical imaging, for example, includes routine asynchronous transfer, mobile ultrasound, multimodal AI, intraoperative guidance, and immersive collaboration. A single maturity label can conceal differences. The tables therefore summarize typical configurations and should be adapted at application level.

Fifth, several added AI and implementation studies use benchmark datasets, observational cohorts, single institutions, or selected populations (36, 37, 67–91, 94). Their findings strengthen the application-level discussion but do not establish broad generalizability. Similarly, successful 5G telemedicine or telesurgery in controlled environments cannot be assumed to transfer directly to routine, resource-constrained, or cross-jurisdictional practice (7, 8, 59, 85, 90, 91).

The field is changing rapidly. Network standardization, regulation, cybersecurity threats, AI methods, and implementation evidence will continue to evolve. The framework should therefore be treated as a revisable governance model rather than a fixed description of future hospitals.

These limitations define the intended use of the synthesis. The framework is designed to organize decisions, expose unsupported assumptions, and specify evidence required before deployment. Its next stage should be empirical validation through use-case-specific pilots, simulation, stakeholder evaluation, implementation research, and comparative health-economic analysis.

### 4.7 Summary and outlook

Future 6G capabilities can contribute to learning hospitals only when they address a demonstrated limitation in an evidence-supported application and operate within an institution capable of governing the associated risks. Research should now move from broad claims about network performance toward comparative, end-to-end evaluation that links technical capability with clinical value, organizational readiness, equity, and sustainable implementation.

## 5 Conclusions

The transition from smart hospitals to learning hospitals should not be defined by adoption of a particular network generation. The evidence reviewed here shows a consistent asymmetry: many healthcare applications—including remote monitoring, telemedicine, imaging, home-based care, logistics, predictive maintenance, environmental sensing, and selected telesurgical procedures—already have clinical, operational, or technical foundations that are substantially more mature than the evidence demonstrating an incremental benefit specifically attributable to 6G. Future 6G should therefore be evaluated against an optimized contemporary baseline rather than treated as the default infrastructure for the next stage of hospital digitalization.

This review proposes an evidence-calibrated decision logic for making that distinction. Hospitals should first define a clinically or operationally meaningful problem and assess the maturity of the application itself. They should then establish what existing wired networks, Wi-Fi, private cellular systems, 5G/5G-Advanced, IoMT, and edge-cloud architectures can already provide. Only when a residual capability gap remains should future 6G be considered as a potential enabling layer. The strongest prospective rationale lies not in higher speed alone, but in combinations of requirements that current systems may struggle to provide consistently at scale, such as predictable end-to-end service, dense mobile-device coordination, multimodal synchronization, integrated sensing and positioning, mobility continuity, and distributed communication-computing orchestration. Whether these capabilities translate into better care remains an empirical question.

The proposed seven-layer architecture, nine-use-case analysis, Evidence Maturity Classification, twelve-domain risk-governance-metric framework, and five-phase implementation roadmap translate this principle into hospital-level decisions. Their common message is that technical performance cannot compensate for deficiencies in clinical validation, interoperability, cybersecurity, human oversight, organizational readiness, equity, or lifecycle sustainability. Scaling should therefore remain conditional on explicit decision gates and on evidence that the intended benefit is reproducible under realistic conditions. The least complex infrastructure capable of meeting the clinical or operational requirement should remain the preferred starting point.

Future research should move from demonstrations of technical possibility toward comparative evaluation. Prospective studies should test advanced connectivity against optimized current alternatives, measure end-to-end rather than isolated network performance, and link technical changes to workflow, safety, clinical outcomes, cost, equity, and environmental burden. High-risk applications will require simulation, multicentre validation, post-deployment surveillance, and robust fallback procedures, while lower-risk operational applications can provide earlier opportunities to generate implementation evidence and strengthen institutional governance capacity.

A 6G-enabled learning hospital is not a hospital in which every application uses 6G. It is a hospital capable of determining when advanced connectivity adds value, when existing infrastructure is sufficient, and when a technology should be modified, paused, or withdrawn. 6G adoption should follow—not precede—evidence of meaningful value, a demonstrated residual capability gap, and governance readiness.
